# Activin Signaling Targeted by Insulin/dFOXO Regulates Aging and Muscle Proteostasis in *Drosophila*

**DOI:** 10.1371/journal.pgen.1003941

**Published:** 2013-11-07

**Authors:** Hua Bai, Ping Kang, Ana Maria Hernandez, Marc Tatar

**Affiliations:** Department of Ecology and Evolutionary Biology, Brown University, Providence, Rhode Island, United States of America; University of California San Francisco, United States of America

## Abstract

Reduced insulin/IGF signaling increases lifespan in many animals. To understand how insulin/IGF mediates lifespan in *Drosophila*, we performed chromatin immunoprecipitation-sequencing analysis with the insulin/IGF regulated transcription factor dFOXO in long-lived insulin/IGF signaling genotypes. Dawdle, an Activin ligand, is bound and repressed by dFOXO when reduced insulin/IGF extends lifespan. Reduced Activin signaling improves performance and protein homeostasis in muscles of aged flies. Activin signaling through the Smad binding element inhibits the transcription of *Autophagy-specific gene 8a (Atg8a)* within muscle, a factor controlling the rate of autophagy. Expression of *Atg8a* within muscle is sufficient to increase lifespan. These data reveal how insulin signaling can regulate aging through control of Activin signaling that in turn controls autophagy, representing a potentially conserved molecular basis for longevity assurance. While reduced Activin within muscle autonomously retards functional aging of this tissue, these effects in muscle also reduce secretion of insulin-like peptides at a distance from the brain. Reduced insulin secretion from the brain may subsequently reinforce longevity assurance through decreased systemic insulin/IGF signaling.

## Author Summary

It is widely known that reduced insulin/IGF signaling slows aging in many contexts. This process requires the forkhead transcription factor (FOXO). FOXO modulates the expression of many genes, and the list of those associated with slow aging is impressive. But there are few data indicating the mechanisms or genes through which FOXO actually slows aging. Here, we identify a novel FOXO target, *dawdle*, the Activin-like ligand in fruit flies. We show that down-regulation of Activin signaling in muscle, but not in adipose tissue, leads to extended lifespan. In part it does so when it alleviates the negative transcriptional repression of its Smox transcription factor (a Smad transcription factor) upon a keystone autophagy gene, *Atg8a*. This double signaling cascade autonomously improves muscle performance (measured at cellular and functional levels) and nonautonomously extends lifespan as it reduces the secretion of insulin peptides from the brain. The work develops the emerging model for interacting autonomous-nonautonomous roles of insulin/IGF signaling as a systems integrative mechanism of aging control.

## Introduction

Reduced insulin/IGF-1 signaling increases the lifespan of nematodes, flies and rodents [1], [2]. In *Caenorhabditis elegans*, mutants in insulin-like receptor *daf-2* live twice as long as wild type [3], [4]. Mutation of insulin receptor *InR* and insulin receptor substrate (*chico*) increase adult lifespan in the fruit fly *Drosophila melanogaster* [5], [6]. It is reported that mice with mutation at the IGF-1 receptor (*Igf1r*) extend lifespan [7], as do mutants of the insulin receptor substrate (Irs2) [8] and of the insulin receptor within adipose tissues [9].

Genetic evidence places the forkhead transcription factor FOXO as the downstream effector of insulin/IGF-1 signaling [3], [10], [11], [12]. Activated insulin/IGF-1 signaling enhances the phosphorylation of FOXO, which is sequestered in the cytoplasm. Conversely, reduced insulin results in FOXO nuclear translocation, which thus promotes or represses the transcription of FOXO target genes [11] (Figure S1A). In *C. elegans* lifespan extension of *daf-2* and *age-1*(PI3 kinase) mutants requires *daf-16*, a FOXO homolog in worms [3]. Recent work likewise shows that FOXO is required for insulin-mediated lifespan extension in *Drosophila* [13], [14]. FOXO also appears to function in human aging where independent studies found polymorphisms of *FoxO3A* to associate with exceptional longevity [15], [16]. Insulin signaling through its control of FOXO is a potentially conserved system to regulate aging but despite this emerging consensus, the proximal targets of insulin/FOXO signaling that orchestrate these mechanisms of longevity assurance are essentially unknown.

One aspect that is clear is that insulin/FOXO signaling operates both nonautonomously and autonomously to control *Drosophila* aging. Systemically reducing insulin signaling by mutations of the insulin receptor (*InR*) and insulin receptor substrate (*chico*) slows the decline in cardiac performance of aging flies while a similar outcome is produced by overexpressing FOXO and PTEN just within cardiomyocytes [17]. Likewise, overexpressing FOXO in muscle maintains muscle protein homeostasis and delays muscle function decline with age while dFOXO expressed in muscles extends lifespan [18], as does expression of dFOXO only from fat body [19], [20]. dFOXO expressed from fat body reduces secretion of systemic insulin-like peptides (DILP2 and DILP5), which are produced predominantly in the brain. dFOXO of *Drosophila* fat body modulates lifespan by inducing fat body *dilp6* transcription, which in turn suppresses neuronal DILP secretion [21]. These findings suggest that insulin/FOXO signaling within some organs controls both the systemic level of circulating DILPs while systemic DILPs regulate somatic maintenance of insulin sensitive tissues. Identifying the FOXO target genes and somatic maintenance pathways in such tissues will elucidate how reduced insulin/IGF-1 assures longevity.

Genome-wide studies with *C. elegans* have been used to probe how *daf-16* controls lifespan in response to insulin signaling. Microarray analyses have identified many mRNA that are affected directly or indirectly by *daf-16*, and reducing some of these genes by RNA interference (RNAi) increases longevity [22], [23]. Chromatin immunoprecipitation (ChIP) and DNA adenine methyltransferase identification (DamID) have been used to identify the direct targets of DAF-16 to clarify which pathways are proximally responsible for the impact of *daf-16* upon aging [24], [25]. Oh et al. [24] thus described 103 genes to be direct targets of *daf-16* in the long-lived *daf-2*(*e1370*) mutant, and three out of 33 tested target genes were found to increase lifespan when gene expressions were reduced by RNAi: *lin-2*, *egl-10* and *sca-1*.

In *Drosophila*, work to date has identified binding targets of dFOXO primarily from wild type adults. Alic et al identified 1423 dFOXO binding sites in wildtype adult female *Drosophila* using a ChIP-on-chip approach [26]. Among 1755 unique genes that are less than 1 kb away from these dFOXO binding sites, about 365 genes are transcriptionally regulated by dFOXO. These targets could potentially modulate many insulin related phenotypes including growth, reproduction, and metabolism, as well as aging.

Here we aim to understand how reduced insulin/IGF-1 signaling extends *Drosophila* lifespan by identifying genes transcriptionally regulated by dFOXO in long-lived insulin signaling mutants. We conducted ChIP analysis with a dFOXO antibody followed by Illumina high-throughput sequencing from *chico* heterozygous mutants, which are long-lived and normal sized, and from adult flies with ablated insulin producing cells (IPCs), which are also long-lived [27]. dFOXO was seen to bind at promoters of 273 genes common to these genotypes, thus providing a candidate set of potential factors in control of aging. Pathways enriched within this set include those for G-proteins, Wnt and Transforming growth factor-beta (TGF-β). We subsequently focused on TGF-β signaling because dFOXO binds in the promoter and represses *dawdle* (*daw*), an Activin-like ligand in TGF-β superfamily. In genetic trials, reducing *daw* or its downstream transcription factor *Smox* increases lifespan, preserves muscle function and reduces poly-ubiquitinated protein accumulation. The muscle specific benefits of activated dFOXO are mediated through the control of autophagy by Smox, which we find to bind and transcriptionally repress *Atg8a/LC3*, a reported longevity assurance gene of *Drosophila* [28]. Expressing *Atg8a* in muscle was also sufficient to increase lifespan. Furthermore, reducing *daw* in muscle decreased DILP2 peptide secretion from the brain while peripheral insulin/IGF signaling was correspondingly reduced. Our results suggest that insulin/IGF signaling controls lifespan in part through dFOXO-mediated repression of muscle Activin signaling and its downstream functions including muscle autophagy, muscle proteostasis and subsequent remote control of systemic insulin/IGF signaling.

## Results

### Activin-like ligand *dawdle* is a direct transcriptional target of dFOXO that regulates longevity

**Figure 1:**
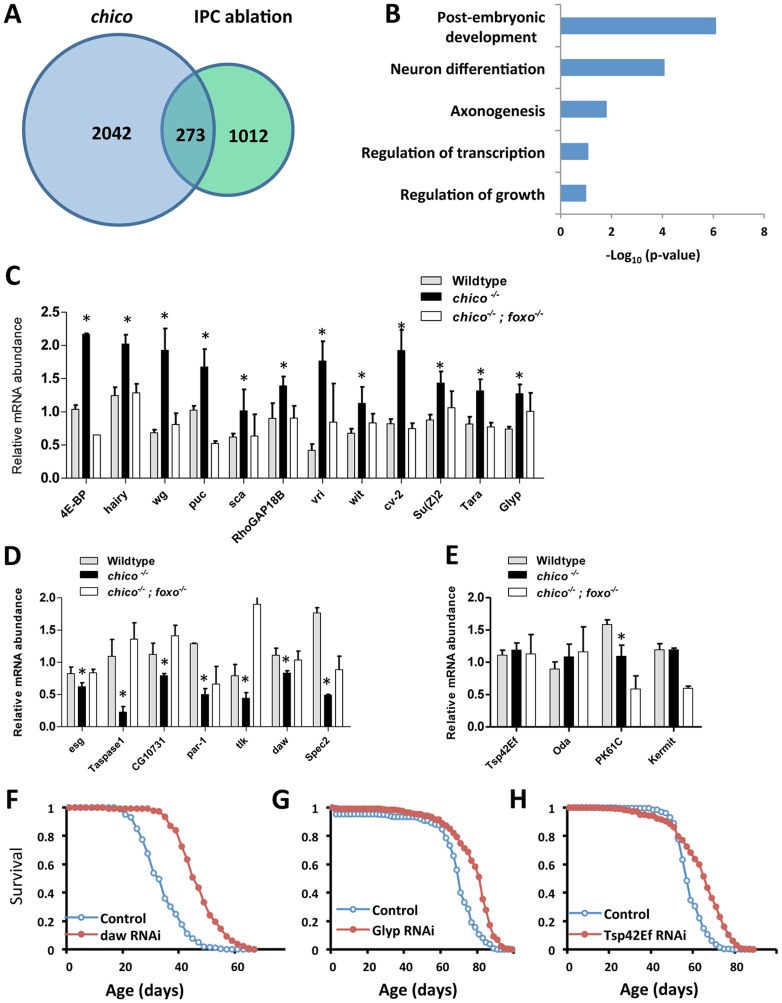
ChIP-Seq to identify dFOXO direct target genes and lifespan screen for 23 selected candidates. (A) Venn diagram to show dFOXO target genes identified in ChIP-Seq analysis. 15-day-old female insulin mutants (*chico ^−/+^* and IPC ablation) were used in ChIP-Seq experiments. dFOXO was enriched at promoters of 273 genes common to these genotypes. (B) Pathway analysis for 273 dFOXO targets, determined by DAVID functional classification. (C–E) Expression analysis of 23 selected dFOXO target genes indicates dFOXO acts as both activator and repressor. Asterisk indicates significant difference between *chico^−/−^* and wildtype (p<0.05); three biological replicates per genotype. (F–H) Lifespan analysis for three dFOXO target genes (*daw*, *Glyp* and *Tsp42Ef*) (Log-rank test, *p*<0.0001). Ubiquitous GeneSwitch (GS)-Gal4 drivers, Tub-GS-Gal4 or Tub-GS-dicer2-Gal4 (with UAS-dicer2 to enhance the knockdown) were used in lifespan screen (lifetable statistics summarized in Table S1).

To understand how dFOXO extends *Drosophila* lifespan we sequenced promoters derived from chromatin-immunoprecipitation with antibody against dFOXO in two genotypes of long-lived flies with reduced insulin signaling. Heterozygotes of *chico^1^* live 36% longer than co-segregating wildtype sibs [13], [29]. Unlike many mutants of the insulin-signaling pathway, *chico* ^+/−^ adults have normal development time, body size and fecundity. Aging is likewise retarded by partially ablating adult IPCs by inducing apoptosis with a cell specific inducible driver (*Dilp2-GeneSwitch-gal4>UAS-reaper*) [27]. We conducted ChIP-Seq analysis from 15-day old female adults from both genetic manipulations. This revealed dFOXO to bind at 1331 and 763 promoter regions (Figure S1B), from *chico* and IPCs ablated flies respectively, corresponding to 2042 and 1012 candidate genes (Figure 1A and Table S7).

We identified 273 genes common to both longevity-assurance genotypes (Figure 1A). Biological functions defined by Gene Ontology (david.abcc.ncifcrf.gov) in this overlapping set include development, growth and neuron differentiation (Figure 1B). Pathway analysis (david.abcc.ncifcrf.gov) revealed enrichment in Wnt and TGF-β signaling (Figure S1C). Corresponding to previous work, we also found significant binding of dFOXO at *puckered* (*puc*) in both longevity assurance genotypes (Table S1). In the JNK signaling pathway, *puc* is a negative regulator of JUN kinase *basket* (*bsk*), and mutation of *puc* extends *Drosophila* lifespan [30].

To determine how candidate dFOXO targets affect longevity we selected 23 genes for further analyses (Figures 1C–1E, Table S1) based on their placement in recognized signaling pathways or because they showed a strong dFOXO binding. dFOXO binding at the promoter of these candidates was verified by ChIP followed with gene specific qPCR. In this analysis dFOXO was significantly enriched at all candidate targets in both insulin mutants when compared to wildtype (Figures S1E–S1F).

To measure the impact of insulin/IGF-1 on candidate transcription we quantified mRNA in adults of wildtype (WT), *chico* null mutant (*chico* ^−/−^) and *chico; foxo* double mutant (*chico ^−/−^; foxo ^−/−^*) (Figures 1C–1E). Transcripts of 12 genes were up-regulated in *chico ^−/−^* relative to wildtype but not in *chico ^−/−^; foxo ^−/−^*, indicating that dFOXO induces these genes. The expression of seven genes was repressed in *chico ^−/−^* relative to wildtype but not in *chico ^−/−^; foxo ^−/−^*, suggesting that dFOXO directly represses these genes. Four genes were not differentially expressed despite their enriched dFOXO binding in the insulin mutants; activated dFOXO may be required but not sufficient to control the expression of these genes. Thus, dFOXO can function as both a transcriptional activator and repressor [26], but this factor may also become poised at genes upon reduced insulin signaling and not affect transcriptional changes until the required co-factors are induced by other signals.

To determine if candidate dFOXO targets contribute to aging regulation we measured lifespan and age-specific mortality when each was reduced by RNAi or over-expressed from transgenes. Cohorts of control and mis-expression genotypes were coisogenic; misexpression was induced only in adults via GeneSwitch (GS)-Gal4 driving either UAS-RNAi or UAS-transgene. The effect of RNAi on lifespan was assessed for all 23 candidates. Knockdown of three genes (*daw*, *Glyp* and *Tsp42Ef*) extended lifespan by consistently reducing age-specific mortality (Figures 1F–1H, Figure S2 and Table S1), while knockdown of 14 genes shortened lifespan (Table S1). Among the candidates whose transcriptions were positively regulated by dFOXO, seven transgenic lines were available to test the effect of overexpression on lifespan; two cases had no effect on lifespan while five cases reduced survival (Table S2).

Among the observed longevity assurance genes, *daw*-RNAi induced by two independent ubiquitous GeneSwitch drivers respectively extended lifespan 12% to 35% (mean lifespan) by consistently reducing mortality rate (Table S1, S3). Dawdle is one of two *Drosophila* Activin-like ligands [31], [32], belonging to the Transforming Growth Factor-β (TGF-β) protein superfamily. To date, *daw* is reported to function in axon guidance [31], [32], cell proliferation and larval brain development [33]. Our results indicate that *daw* acts as a downstream target of dFOXO to modulate lifespan, suggesting that the Activin branch of TGF- β signaling may participate in control of aging.

### Activin signaling within muscle regulates *Drosophila* lifespan

**Figure 2:**
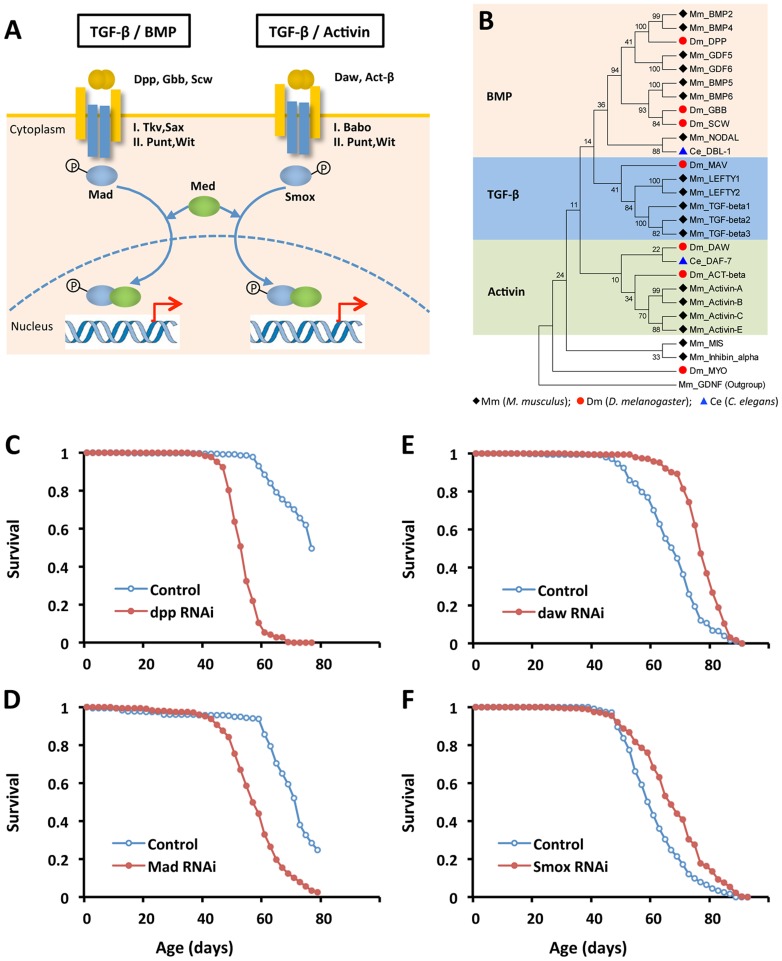
Reducing Activin signaling, but not BMP signaling prolongs lifespan in *Drosophila*. (A) Schematic showing distinct TGF-β pathways in *Drosophila*: BMP and Activin. (B) Phylogenetic analysis of TGF-β ligands from worm, fly and mouse. Ligand sequences were retrieved from Flybase, Wormbase and Genebank, respectively. The phylogeny was constructed using MEGA 5.0. (C–F) Lifespan analysis of TGF-β pathways in *Drosophila* using ubiquitous GeneSwitch (GS)-Gal4 drivers, Tub-GS-Gal4 or da-GS-gal4 (Log-rank test, *p*<0.0001). See Table S3 for survival analysis.

*Drosophila* has two TGF-β ligand subfamilies: bone morphogenetic proteins (BMP) (ligands: Dpp, Gbb and Scw) and Activin (ligands: Daw and Act-β). These ligands signal through subfamily-specific Type I receptors (Tkv and Sax for BMP, Babo for Activin) and shared Type II receptors (Punt, Wit). BMP-like ligands and Activin-like ligands activate distinct downstream signaling cascades leading respectively to phosphorylation of the Smad transcription factors Mad and Smox (Figure 2A) [34].

Since *daw*-RNAi increases lifespan, we determined whether other elements of either TGF-β pathway could likewise control aging (Figures 2C–2F and Figure S3). RNAi for *Smox*, the Activin associated Smad transcription factor, extended lifespan 10% (Figure 2F). RNAi for Activin receptor *babo* and the Activin-like ligand *Act-β* did not affect survival. Repressing the BMP branch of TGF-β signaling via RNAi for *dpp*, *gbb*, *Mad* and *Tkv* consistently reduced survival (Table S3). Ubiquitously overexpressing genes in either Activin or BMP subfamily shortened lifespan (data not shown), as did RNAi for co-Smad (*Med*), the shared Type-II receptor (*Punt* and *Wit*) and two other TGF- β ligands (*Myo* and *Mav*) (Table S3).

The TGF-β signaling pathways of *Drosophila* are homologous to *C. elegans* TGF-β/dauer and Sma/Mab. Recent reports clarify that the TGF-β/dauer pathway can regulate somatic aging, while the Sma/Mab pathway appears to modulate reproductive aging [35], [36]. We performed a phylogenetic analysis on TGF-β ligands of *C. elegans*, *Drosophila* and mouse (Figure 2B). Similar to previous published phylogenetic analysis [37], [38], we found that the TGF-β/dauer ligand of *C. elegans*, DAF-7, is closely related to Activin-like ligands in *Drosophila* (Daw and Activin-β) and mouse (Activin-A, B, C and E), while the Sma/Mab ligand in *C. elegans*, DBL-1, is similar to BMP-like ligands in *Drosophila* and mouse. Together these results suggest that Activin may be a conserved longevity pathway.

**Figure 3:**
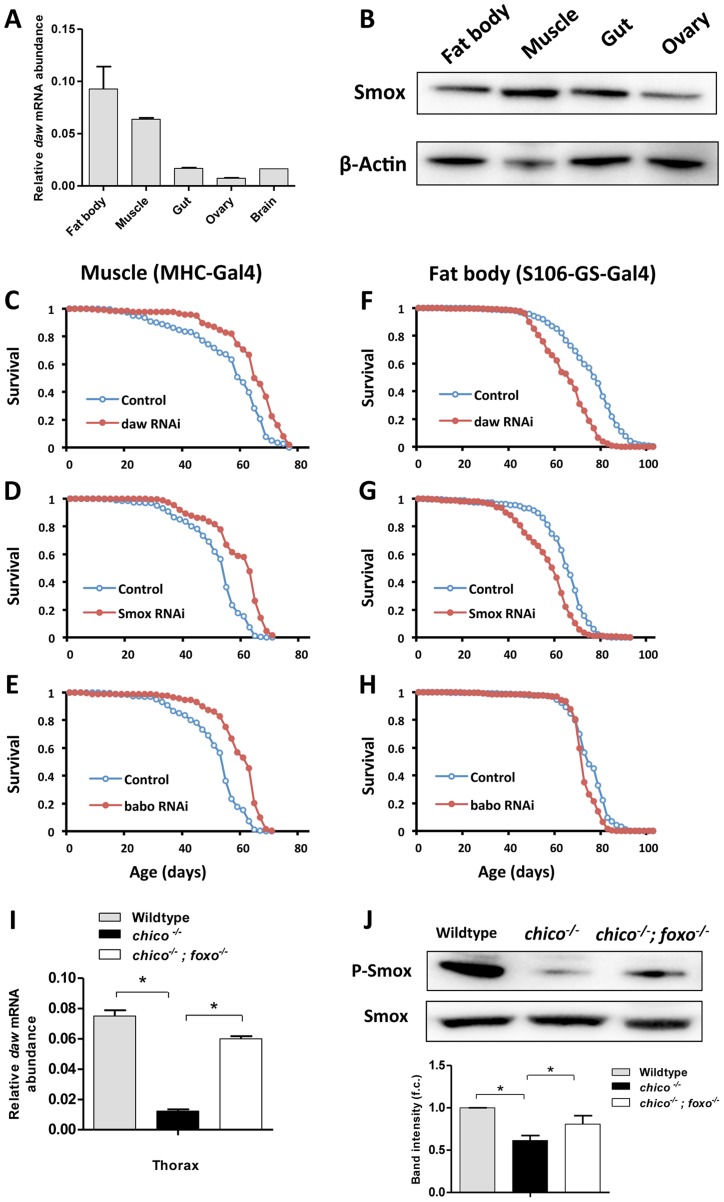
Inactivation of genes in Activin signaling (*daw*, *Smox* and *babo*) in muscle, but not in fat body extended lifespan. (A) Tissue-specific gene expression pattern of *daw*. (B) Tissue-specific distribution of transcription factor Smox using 7-day-old *Oregon R* females. (C–E) Lifespan analysis of Activin signaling using muscle-specific Gal4 driver (MHC-Gal4). Lifespan was extended by inactivating Activin genes (*daw*, *Smox* and *babo*) in muscle (Log-rank test, *p*<0.0001). (F–H) Lifespan analysis of Activin signaling using adult fat body-specific Gal4 driver (S106-GS-Gal4). Fat body-specific inactivation of Activin genes (*daw* and *Smox*) shortens lifespan (Log-rank test, *p*<0.0001). See Table S4 for survival analysis. (I, J) mRNA expression of *daw* and phosphorylation of Smox are down-regulated by *chico* mutation and rescued by mutation of dFOXO. Muscle and fat body were dissected from 7-day-old female wildtype, *chico^−/−^* and *chico;foxo* double mutants. Band intensity was quantified using Bio-Rad Image Lab software. The average band intensity from four independent experiments is shown. Asterisk indicates significant difference between treatment and control (*p*<0.05).

To understand how Activin regulates *Drosophila* aging we determined which tissues produced this control. *daw* mRNA is highly expressed in muscle and fat body, a tissue with both liver and adipose-like activities (Figure 3A). Smox protein is more widely distributed (Figure 3B). To assess the role of Activin in muscle and fat body we knocked down *daw*, *Smox* and *babo* with tissue-specific drivers. Lifespan was extended by inactivating each of these genes in muscle, but not in fat body (Figures 3C–3H, Figure S4 and Table S4). Since *daw* is a dFOXO downstream target that is down-regulated in *chico* mutants (Figure 1D), we determined whether reduced insulin/IGF-1 signaling modulates Activin within muscle. *chico* mutants showed reduced *daw* mRNA sampled from thorax (containing mostly flight muscle), and this effect was reversed in *chico; foxo* double mutants (Figure 3I). Furthermore, Smox protein was less phosophorylated in *chico* mutants (Figure 3J). Insulin/IGF-1 signaling, through dFOXO, thus appears to modulate muscle Activin signaling, which in turn is sufficient to regulate longevity.

### Muscle Activin signaling regulates proteostasis and autophagy

Muscle performance in many animals declines in parallel to the accumulation of misfolded protein aggregates [39]. Insulin/IGF-1 signaling in *Drosophila* may affect this process since over-expressing dFOXO in *Drosophila* muscle slows the aggregate accumulation and promotes macroautophagy [18]. Here we determine whether dFOXO mediates its effects on muscle proteostasis and function through its control of Activin.

**Figure 4:**
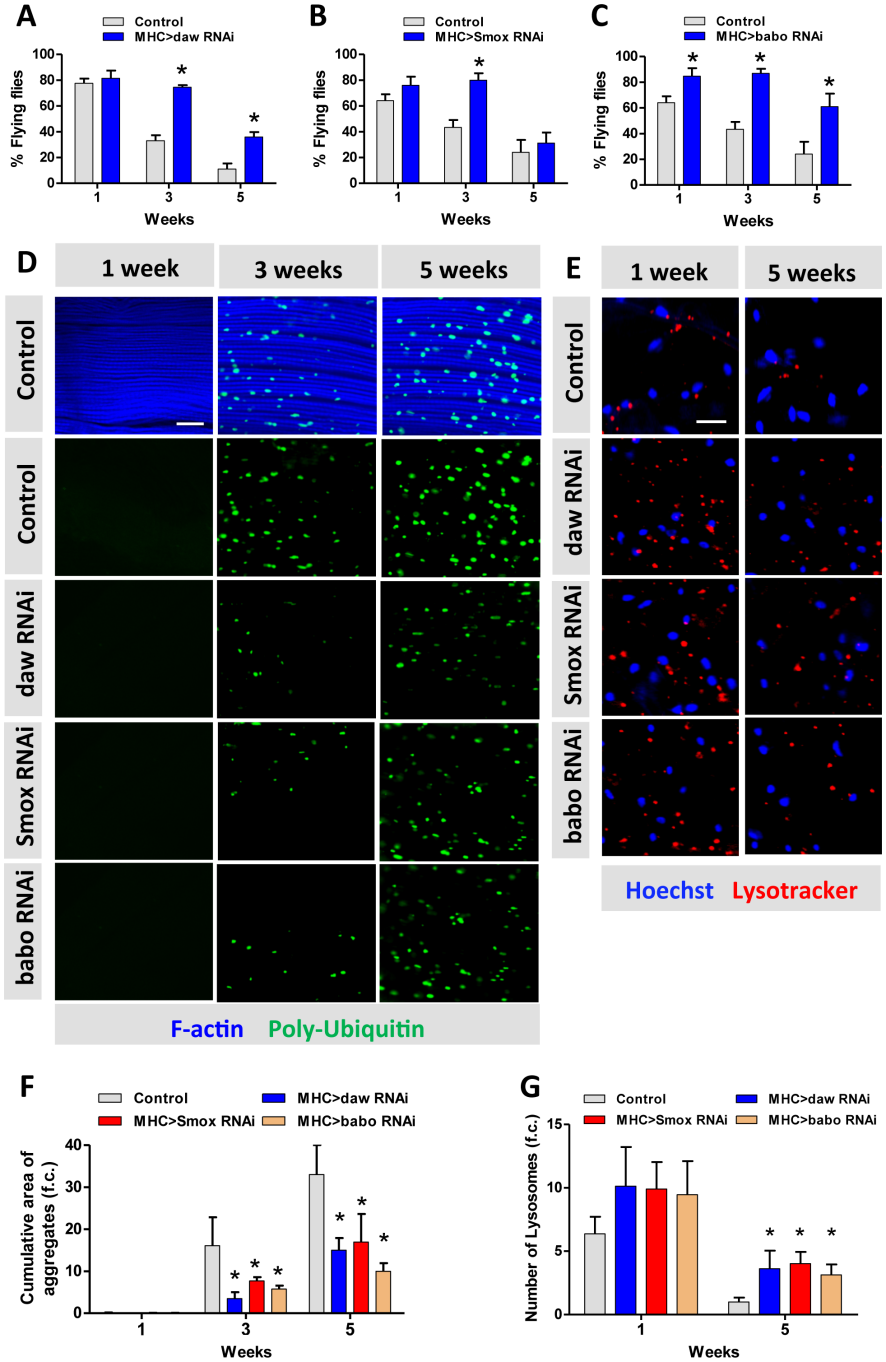
Activin signaling regulates muscle aging and proteostasis. (A–C) Decline of flight with age is delayed in *daw*, *Smox* and *babo* RNAi flies. 40 females were scored for each genotype at each time point. Flying ability was measured at one week, three weeks and five weeks. (D) Poly-Ubiquitin-positive protein aggregates are reduced at old age in *daw*, *Smox* and *babo* RNAi flies. Aggregates were visualized with Poly-Ubiquitin FK2 antibody at one week, three weeks and five weeks. Scale bar: 20 µm. (E) RNAi for *daw*, *Smox* and *babo* preserves the decline of lysotracker-positive organelles (lysosomes). Scale bar: 20 µm. (F) Quantification of the cumulative area of protein aggregates for Figure 4D (n = 20). (G) Quantification of the number of lysotracker-positive stain for Figure 4E (n = 10). Asterisk indicates significant difference between treatment and control (*p*<0.05).

Experimentally reducing Activin prevents the decline of muscle function with age. Flight activity typically declines in aging flies [18], as it does in our wildtype control (Figures 4A–4C). RNAi against the Activin factors *daw*, *Smox* and *babo* each retarded this decline (Figures 4A–4C). Likewise, the ability to climb at advanced ages was preserved in *daw* RNAi flies relative to wildtype (Figure S6D). Progression of these composite movement traits was associated with changes in protein aggregates within muscle. Aggregates visualized with Poly-Ubiquitin FK2 antibody increase with age in wildtype muscle, but this change was significantly delayed by muscle specific RNAi against *daw*, *Smox* or *babo* (Figure 4D, F).

**Figure 5:**
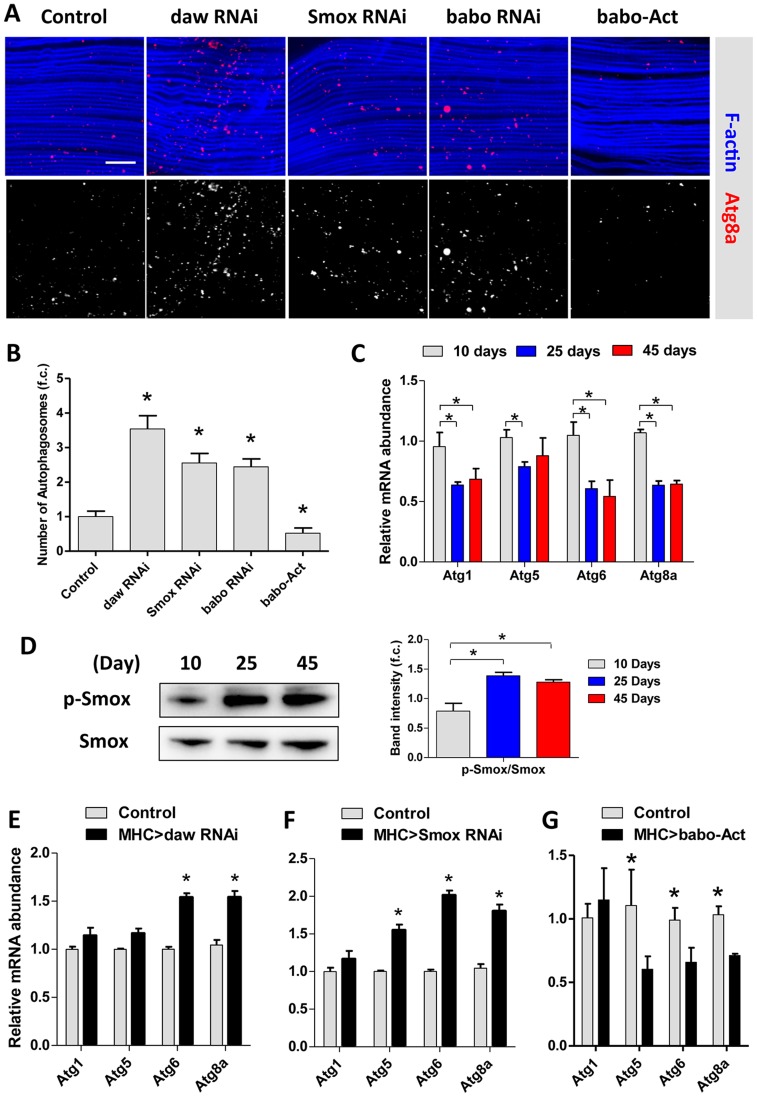
Activin regulates muscle autophagy. (A) Autophagosomes indicated using an *Atg8a*-Cherry reporter in Activin RNAi flies or *babo* over-expressing (*babo-Act*) flies. 3-day old females. (B) Quantification of autophagosomes for Figure 5A (n = 20). (C) mRNA expression of autophagy genes (*Atg1*, *Atg5*, *Atg6* and *Atg8a*) in aging muscle, at 10 days, 25 days and 45 days. (D) Phosphorylation of Smox in muscle increases with age. The average band intensity from three independent experiments was quantified using Image Lab software. (E) Inactivation of *daw* in muscle up-regulates autophagy gene expression. (F) RNAi against *Smox* in muscle up-regulates autophagy gene expression. (G) Constitutive activation of *babo* (*babo-Act*) in muscle inhibits autophagy gene expression. Asterisk indicates significant difference between treatment and control (*p*<0.05).

Macroautophagy modulates protein aggregate accumulation [40]. We used two markers of lysosome/autophagy activity, lysotracker and cherry-tagged-Atg8a (homolog of LC3), to determine if Activin regulates muscle proteostasis through macroautophagy. The intensity of lysosome markers decreased with age in wildtype flight muscle, but was maintained in aged muscle expressing RNAi for *daw*, *Smox*, or *babo* (Figure 4E, quantified in Figure 4G). We likewise observed more autophagosomes in flight muscle with inactivated TGF- β/Activin signaling (via RNAi for *daw*, *Smox*, or *babo*) (Figure 5A). In contrast, constitutively activated Activin signaling (via overexpressing *babo*-Act) reduced the number of autophagosomes (Figure 5A, 5B). Since Activin signaling is transcriptionally regulated by dFOXO via *daw*, these results may explain reported associations between reduced insulin signaling and elevated autophagy [18]. Reduced insulin signaling represses Activin, which in turn releases repression of autophagy and thereby reduces accumulation of protein aggregates.

### Activin signaling represses autophagy via transcriptional regulation on Atg8a

*Drosophila* encode 18 autophagy genes [41]. Many of these are less expressed in aged flies (Figure 5C) [18]. Since reduced *Smox* mRNA produces elevated autophagy in aged muscle, we studied the phosphorylation of this transcription factor in old flies. Smox phosphorylation is increased in aging muscle (Figure 5D), suggesting that Activin may be a negative regulator of *Atg* gene expression. Indeed, *Atg6* and *Atg8a* mRNA were increased when *daw* and *Smox* were reduced in muscle (Figure 5E, 5F), while mRNA of *Atg5*, *Atg6* and *Atg8a* were reduced by over-expressing constitutively active form of the *babo* receptor (Figure 5G).

**Figure 6:**
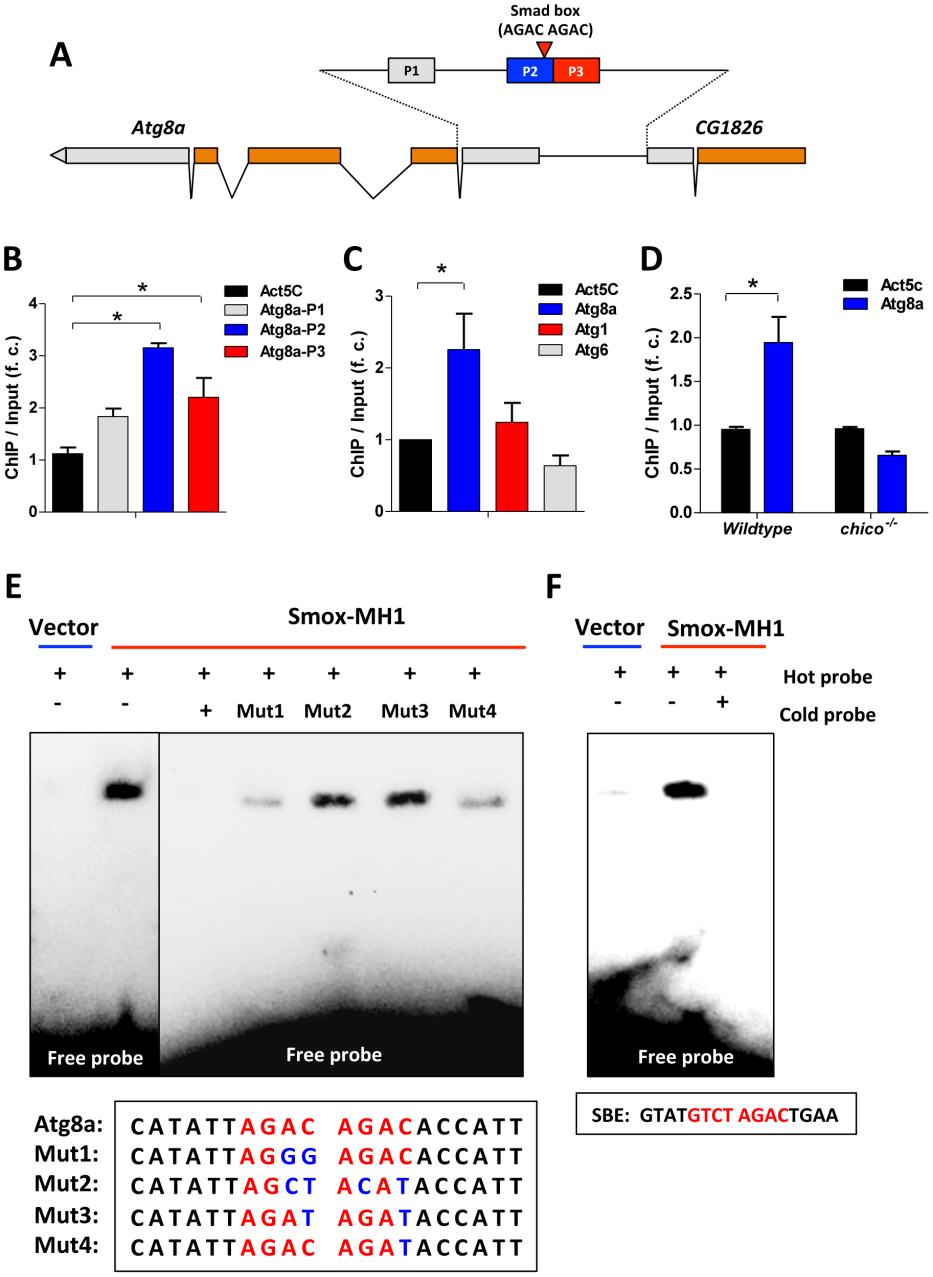
Activin signaling represses autophagy via transcriptional regulation on Atg8a. (A) Schematic of *Atg8a* genomic region (Smad box and ChIP-PCR target regions (P1–P3) are shown). Gray bar represents UTR and orange bar represents exon. (B) ChIP-PCR shows Smox binds to the promoter of *Atg8a* with binding enrichment calculated as the fold change of ChIP DNA vs. input DNA. The binding to the coding region of Actin gene (Act5C) was used as a negative control. (C) Smox binds to the promoter of *Atg8a*, but not *Atg1* and *Atg6*. The primers targeting the promoter regions containing putative Smad box in *Atg8a*, *Atg1* and *Atg6* were used in ChIP-PCR. (D) The binding of Smox to *Atg8a* promoter is abolished by *chico* mutation. Asterisk indicates significant difference between treatment and control (*p*<0.05). (E). EMSA analysis reveals that recombinant Smox protein (MH1-DNA binding domain) binds to Smad binding element (AGAC AGAC) located in *Atg8a* promoter. Biotin-labeled *Atg8a* oligonucleotide probe (5′- CATATTAGAC AGACACCATT -3′) and its mutated forms are labeled with biotin. Halo-tagged Smox-MH1 DNA binding domain (amino acids 1–140) are expressed in *E. coli* and purified before used in EMSA analysis. (F) Recombinant Smox protein can also bind to mammalian SBE (GTATGTCT AGACTGAA).

*Drosophila* Smox protein is homologous to vertebrate Smad2 and Smad3 transcription factors. Human Smad3 protein recognizes the consensus sequence GTCTAGAC [42], although a single copy of the Smad box (GTCT) is also reported to support Smad3 binding at the MH1 domain [43]. We searched the promoter regions of *Atg8a* and identified at least two adjacent Smad boxes located within *Atg8a* (Figure 6A). ChIP-PCR with affinity-purified Smox antibody showed that Smox binds to the promoter region of *Atg8a* (Figure 6B), but not *Atg1* and *Atg6* (Figure 6C). In contrast to Smox, dFOXO does not bind to the promoter of *Atg8a* (Figure S6E). This is unlike mammalian FoxO3 which induces autophagy by directly binding to the promoters of *LC3b, Gabarapl1, and Atg12l* in C2C12 myotubes [44]. Consistent with our model for negative regulation on Activin signaling by activated dFOXO, *chico ^−/−^* inhibits Smox binding at *Atg8a* (Figure 6D).

An electrophoretic mobility shift assay (EMSA) confirms that Smox binds directly within *Atg8a* promoter. We expressed and purified a recombinant protein of the Smox-MH1 DNA binding domain (amino acids 1–140) and measured its interaction with biotin-labeled *Atg8a* oligonucleotide probes containing Smad box (5′-AGAC AGAC-3′). Smox-MH1 strongly bound to the *Atg8a* probe, and this interaction was blocked by addition of unlabeled wildtype cold probes (Figure 6E). To define the required sequences of this Smad box (AGAC) we competed labeled wildtype probe with mutated cold probes. Unlabeled cold probes with mutations in both Smad boxes (Mut2 and Mut3) did not compete with the wildtype binding, but cold probe mutants for only a single only Smad box (Mut1 and Mut4) retained some competitive ability (Figure 6E). Furthermore, *in vitro* expressed Smox-MH1 also binds to the oligonucleotide probe for the vertebrate Smad binding element (5′-GTCT AGAC-3′) (Figure 6F). Together these data identify an invertebrate Smad binding element (AGAC AGAC) in the promoter region of the autophagy gene *Atg8a*. This Smad binding element contains a direct repeat of two Smad boxes (AGAC). Upon activation, Smox, the *Drosophila* homologue of Smad2/3, binds to the Smad box located within the promoter of *Atg8a*. Activin signaling represses autophagy via direct transcriptional regulation on the key autophagy gene *Atg8a*.

**Figure 7:**
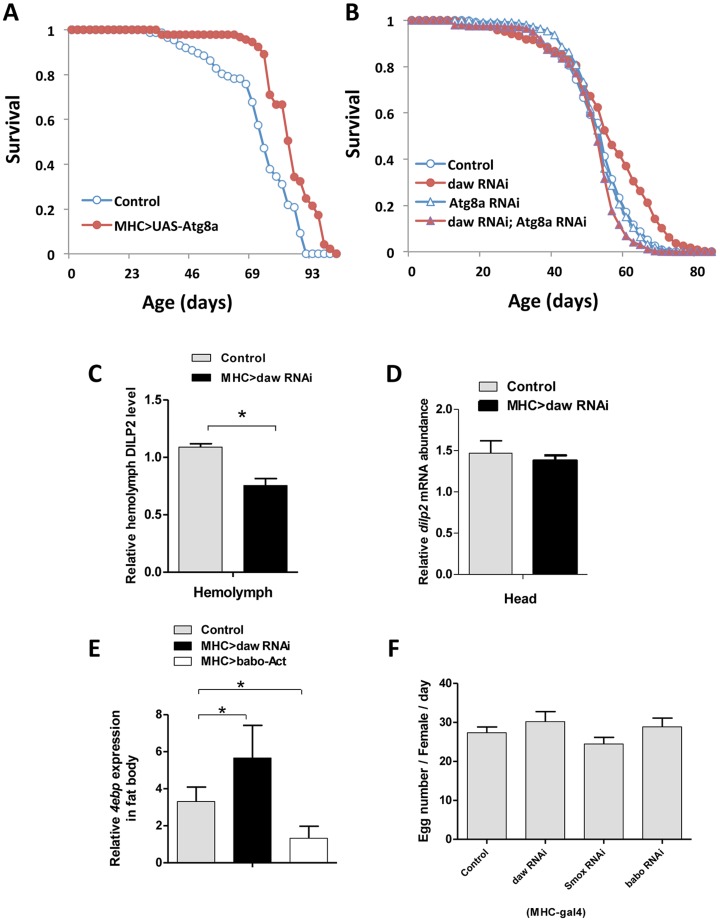
Muscle Activin signaling regulates longevity through *Atg8a* and remotely controls brain insulin secretion. (A) Lifespan analysis of muscle-specific *Atg8a* overexpression. (B) Genetic epistasis between *daw* and *Atg8a* in muscle (MHC-Gal4). Simultaneous expression of RNAi for *daw* and *Atg8a* blocks the longevity benefit of *daw* RNAi alone (while *Atg8a* RNAi alone does not affect survival). See Table S5, S6 for survival analysis. (C, D) Muscle-specific *daw* RNAi reduces circulating DILP2 level, but has no effects on *dilp2* mRNA expression in the head. (E) *4ebp* mRNA expression in fat body is regulated by muscle Activin signaling. *4ebp* mRNA is elevated in fat body when *daw* is reduced in muscle, while it is repressed when muscle *babo* is induced. (F) Female fecundity is not affected by reducing muscle Activin signaling. Asterisk indicates significant difference between treatment and control (*p*<0.05).

To test whether increasing *Atg8a* expression within muscle is sufficient to promote lifespan, we over-expressing *Atg8a* using a muscle-specific driver (MHC-Gal4). Lifespan was modestly but significantly increased, suggesting that *Atg8a* gene is a specific instance of a longevity assurance genes that that functions through muscle downstream of Activin signaling (Figure 7A, Table S4). To further examine whether *Atg8a* is required for Activin-mediated lifespan extension, we silenced both *Atg8a* and *daw* using muscle-specific RNAi (Figure 7B, Figure S7, Table S5). Lifespan extension when *daw* RNAi was expressed in muscle was rescued when *Atg8a* was simultaneously reduced by RNAi in this tissue, indicating Activin regulates longevity through muscle *Atg8a*.

### Muscle Activin signaling remotely controls brain insulin secretion

Previous studies from *C. elegans* recognize cross-talk between insulin/IGF-1 and TGF-β pathways [36], [45]. DAF-16 is nuclear localized in many mutants of the TGF-β/dauer pathway [36]. To determine if muscle Activin signaling affects *Drosophila* lifespan through systemic insulin/IGF-1signaling we measured circulating insulin-like peptides in adults with tissue specific *daw*-RNAi. Knockdown of *daw* in muscle reduced the level of hemolymph DILP2 (Figure 7C), while *dilp2* mRNA remained constant (Figure 7D), suggesting the daw specifically modulates DILP2 secretion. In contrast, knockdown of *daw* in fat body increased the level of circulating DILP2 (Figure S6F). These contrasting tissue associated changes correspond to the observed effects upon lifespan when *daw* is reduced in each tissue (Figure 3C–3H). Notably, *dilp2* mRNA is reduced in other tissue-limited genetic manipulations that extend lifespan [19], [46], and knockout of the *dilp2* locus is sufficient to extend lifespan [47]. We now see that reducing muscle Activin signaling via *daw* RNAi also remotely controls DILP2 secretion from the brain. This is sufficient to decrease systemic insulin signaling because insulin/FOXO sensitive *4ebp* mRNA is elevated in peripheral tissues (e.g. fat body) (Figure 7E).

Unlike the consistent response of *4eBP* in longevity mutants with reduced insulin, some but not all insulin/IGF pathway mutants have reduced fecundity. Here we found normal fecundity in females with reduced muscle Activin signaling (Figure 7F), suggesting the effects of muscle Activin on lifespan are not mediated through trade-off between longevity and reproduction.

## Discussion

Insulin/IGF-1 signaling modulates longevity in many animals. Genetic analysis in *C. elegans* and *Drosophila* shows that insulin/IGF-1 signaling requires the DAF-16/FOXO transcription factor to extend lifespan, while in humans several polymorphisms of FoxO3A are associated with exceptional longevity [15], [16]. Although many downstream effectors of FOXO have been identified through genome-wide studies [22], [24], [25], [26], the targets of FOXO responsible for longevity assurance upon reduced insulin signaling are largely unknown [24]. Here we found 273 genes targeted by *Drosophila* FOXO using ChIP-Seq with two long-lived insulin mutant genotypes. We focused on *daw*, an Activin ligand, which is transcriptionally repressed by FOXO upon reduced insulin/IGF signaling. Inactivation of *daw* and of its downstream signaling partners *babo* and *Smox* extend lifespan. These results are reminiscent of observations from *C. elegans* where reduced TGF-β/dauer signaling extends longevity [36]. Notably, the lifespan extension of TGF-β/dauer mutants (e.g. *daf-7 (e1372)* mutants) can be suppressed by *daf-16* mutants, suggesting that TGF-β signaling intersects with the insulin/IGF-1 pathway for longevity in *C. elegans* [36]. In our phylogenetic analysis, DAF-7, Daw and mammalian Activin-like proteins share common ancestry. Activin signaling, in response to insulin/IGF-1, may thus represent a taxonomically conserved longevity assurance pathway.

Longevity benefits of reduced Activin (TGF-β/dauer) in *C. elegans* were resolved only when the matricide or ‘bagging’ (due to progeny hatching within the mother) was prevented by treating *daf-7(e1372)* mutants with 5-fluorodeoxyuridine (FUdR) to block progeny development [36]. This approach made it possible to distinguish the role of Activin in somatic aging from the previously recognized influence of BMP (Sma/Mab signaling) upon reproductive aging in *C. elegans* [35], [48]. Activin, of course, is a somatically expressed regulatory hormone of mammalian menstrual cycles that induces follicle-stimulating hormone (FSH) in the pituitary gland. In young females, FSH is suppressed within a cycle when maturing follicles secrete the related TGF-β hormone Inhibin [49]. In mammalian reproductive aging, the effect of Activin in the pituitary becomes unopposed as the stock of primary follicles declines, thus inducing elevated production of FSH. We now find that reduced Activin but not BMP signaling favors somatic persistence in *Drosophila*. These parallels between reproductive and somatic aging among invertebrate models and humans suggest that unopposed Activin signaling is pro-aging while favoring reproduction.

Reduced insulin/IGF signaling extends lifespan through interacting autonomous and non-autonomous actions. Reducing IIS in some distal tissues has been shown to slow aging because this reduces insulin secretion from a few neurons: reducing IIS by increasing dFOXO in fat body or muscle extends *Drosophila* fly lifespan while decreasing IPC production of systemically secreted DILP2 [18], [19]. Here we identify Activin as a direct, downstream target of insulin/dFOXO signaling within muscles that has the capacity to non-autonomously regulate lifespan. Knockdown of Activin in muscle but not in fat body is sufficient to prolong lifespan. RNAi for muscle Activin signaling led to decreased circulating DILP2 and increased peripheral insulin signaling. Muscle is thus proposed to produce a signaling factor, a myokine, which impacts organism-wide aging and metabolism [18], [50], [51] (Figure S8).

Aging muscle may produce different myokine-like signals in response to their physiological state. Aged muscles degenerate in many ways including changes in composition, mitochondria, regenerative potential and within-cell protein homeostasis [52]. Protein homeostasis is normally maintained, at least in part, by autophagy [40], [53]. Loss of macroautophagy and chaperone-mediated autophagy with age will accelerate the accumulation of damaged proteins [54]. Expression of *Atg8a* in *Drosophila* CNS is reported to extend lifespan by 56% [28], while recent studies find elevated autophagy in long-lived mutants including those of the insulin/IGF-1 signaling pathway [18], [55], [56]. Our results now show that insulin/IGF signaling can regulate autophagy through its control of Activin via dFOXO. Poly-ubiquitinated proteins accumulate in aging *Drosophila* while lysosome activity and macroautophagy decline. Muscle performance with age (flight, climbing) was preserved by inactivating Activin within this tissue. This genetic treatment also reduced the accumulation of protein aggregates. These effects are mediated by blocking the transcription factor Smox, which otherwise represses *Atg8a*. Smox directly regulates *Atg8a* through its conserved Smad binding motif (AGAC AGAC). These results, however, contrast with an observation where TGF-β1 promotes autophagy in mouse mesangial cells [57].

Insulin/IGF-1 signaling is a widely conserved longevity assurance pathway. Our data indicate that reduced insulin/IGF-1 retards aging at least in part through its FOXO-mediated control of Activin. Furthermore, affecting Activin only in muscle is sufficient to slow its functional decline as well as to extend lifespan. Autophagy within aging muscle controls these outcomes, and we now find that Activin directly regulates autophagy through Smox-mediated repression of *Atg8a*. If extrapolated to mammals, pharmaceutical manipulations of Activin may reduce age-dependent muscle pathology associated with impaired autophagy, and potentially increase healthy and total lifespan through beneficial signaling derived from such preserved tissue.

## Materials and Methods

### Fly husbandry and stocks

Flies were reared and maintained at 25°C, 40% relative humidity and 12-hour light/dark. Adults were maintained upon agar-based diet with cornmeal (0.8%), sugar (10%), and yeast (8% unless otherwise noted). RU486 (mifepristone, Sigma, St. Louis, MO, USA) used to activate GeneSwitch-Gal4 was dissolved in ethanol to a concentration of 200 µM and added to the food.

Fly stocks included: *MHC-Gal4* [18], *Tub-GS-Gal4* [58]; *da-GS-Gal4* [59]; *S106-GS-Gal4* [58]; *dilp2-GS-Gal4* (provided by H. Jasper); *dilp2-GS-Gal4,UAS-dicer2* (provided by S. Helfand). RNAi lines for dFOXO target genes and TGF-β pathway are from Bloomington stock center (TRiP line) and Vienna Drosophila RNAi Center (see the supplemental Table for the stock number). UAS-lines are: *UAS-hairy* [60], *UAS-puc* [61], *UAS-RhoGAP18B* [62], *UAS-vri* [63], *UAS-wit* [64], *UAS-cv-2* [65], *UAS-babo-Act* (also known as UAS-babo*1A2) [66], *UAS-Atg8-Cherry* [67]. MHC-Gal4 is a constitutive muscle-specific driver, with expression restricted to the thoracic region and legs (data not shown).

*Chico* mutants were made in our lab as describe previously [13], [29]: *y^1^; cn^1^; ry^506^* (wildtype), *y^1^; cn^1^ chico^1^/cn^1^; ry^506^* (*chico^−/+^*), *y^1^; cn^1^ chico^1^/cn^1^ chico^1^; ry^506^* (*chico^−/−^*), *y^1^; cn^1^ chico^1^/cn^1^ chico^1^; foxo^21^ ry^506^* (*chico^−/−^, foxo^−/−^*). Adult on-set IPC ablation flies were made by crossing *Dilp2-GS-Gal4* to *UAS-rpr* and inducing the cell death in IPC cells by feeding flies with RU486 for 15 days.

### ChIP-Seq, ChIP-PCR and data analysis

Two insulin mutants were used in ChIP-Seq experiments, *chico* ^−/+^ and IPC ablation. Chromatin immunoprecipitation (ChIP) was performed according to previously published methods with modification [68], [69], [70]. About 200–250 adult females (∼200 mg) at the age of 15-day-old were pooled for each ChIP sample. Two biological replicates were prepared for each genotype. Flies were homogenized and cross-linked in 1× PBS containing 1% formaldehyde. The fly lysate were sonicated using a Branson 450 sonicator to break down the chromatin into a pool of DNA fragment with average size of 500 bp. Immunoprecipitation was performed using Dynal protean A beads (Invitrogen, Grand Island, NY, USA) and affinity purified anti-dFOXO antibody made in our laboratory. Following the wash with LiCl and TE buffer, the DNA-protein complex was eluted from the Dynal beads and reverse cross-linked. After Proteinase K digestion, dFOXO-bound DNA fragments were purified and diluted in Tris-HCl buffer. About 20 ng of ChIP DNA (dFOXO-bound DNA) and input DNA (DNA sample before the immunoprecipitation) were used in library preparation following the methods described in [71]. The libraries were then size-selected (150 bp-350 bp) and purified by agarose gel, and subjected to the Illumina Genome Analyzer IIx Sequencer (Illumina, San Diego, CA, USA).

To map the dFOXO binding sites, we pooled the raw reads (about 20 million reads per sample) from two replicates into one data file and aligned it to *Drosophila* reference genome using Bowtie short read aligner [72]. About 70% of raw reads have at least one alignment. The enrichment of dFOXO binding between ChIP DNA and input DNA was determined using peak calling package PeakSeq [73]. Enriched regions with FDR of 0.01 were selected. Target genes, which were detected 5 kb away from the center of the binding sites, were also obtained. The ChIP-Seq raw data are archived at NCBI GEO with Accession # GSE44686.

For ChIP-PCR analysis, the binding enrichment was calculated as the fold change of ChIP DNA versus input DNA. The binding to the coding region of Actin gene (*Act5C*) and *sry* genomic region were used as negative controls.

### Pathway and motif analysis

The DAVID functional classification tool was used for pathway and molecular function analysis on the dFOXO target genes [74]. Genomic sequence near the dFOXO binding region (∼200 bp) was downloaded from the Flybase (http://flybase.org/) and *de novo* motif analysis was performed using MEME Suite [75].

### Quantitative RT–PCR

Total RNA was extracted from 10 whole flies or from tissue of 15 flies in Trizol reagent (Invitrogen, Grand Island, NY, USA). DNase-treated total RNA was quantified with a NanoDrop ND-1000. About 50–100 ng of total RNA was used for quantification with SuperScript One-Step RT-PCR reagent (Invitrogen, Grand Island, NY, USA) and measured on an ABI prism 7300 Sequence Detection System (Applied Biosystems, Carlsbad, CA, USA). Three biological replicates were used for each experimental treatment. mRNA abundance of each gene was normalized relative to ribosomal protein L32 (*RpL32*, also known as *rp49*) by the method of comparative C_T_. Primer sequences are shown in Table S8.

### Phylogenetic analysis

Full length TGF-β ligands from worm, fly and mouse were aligned in ClustalW. From the alignments, a phylogenetic tree was constructed using MEGA 5.0 [76], according to the neighbor-joining method with a bootstrap test calculated with 2000 replicates and a poisson correction model. Mouse Glial cell line-derived neurotrophic factor (GDNF) was used as the out-group.

### Demography and survival analysis

Two to three-day-old female adult flies were collected with light CO_2_ anesthesia and pooled in 1 L demography cages at a density of 100 to 125 flies per cages. Three independent cages were initiated per genotype. Food vials with media containing vehicle only or RU486 were changed every two days, at which time dead flies were removed and recorded. Survival analysis was conducted with JMP statistical software with data from replicate cages combined. Survival distributions were compared by the Log-Rank test. Cox proportional hazard survival analysis was used to assess how reduced *daw* and *Atg8a* interacted to affect mortality.

### Flying and climbing assays

Flying and climbing assays were scored as described in [18]. In the flying assay, flies were released at the top of a 250 ml cylinder (about 30 cm long). The number of flies that didn't fall straight to the bottom of the cylinder was recorded. A total of 40 females were scored for each genotype.

In the climbing assay (also known as negative geotaxis assay), flies were first tapped down to the bottom of a standard (empty) food vial, and the percentage of flies that climbed up 8 cm within 20 seconds was recorded. A total of 80 females (10 flies per vial) were scored for each genotype.

### Immunostaining and imaging

Antibodies for immunostaining included: anti-polyubiquitin FK2 (1∶200) (Assay Designs/Enzo Life Sciences, Farmingdale, NY, USA), and anti-rabbit IgG-DyLight 488 (1∶300) (Jackson ImmunoResearch, West Grove, PA, USA). F-actin was visualized by Alexa Fluor 488-conjugated Phalloidin (Invitrogen, Grand Island, NY, USA). Lysosome was monitored by LysoTracker Red DND-99 at the concentration of 100 nM (Invitrogen, Grand Island, NY, USA). DNA was stained with Hoechst 33342 (1 µg/ml) (Invitrogen, Grand Island, NY, USA). Samples were processed as described in [18], and imaged with a Leica SP2 laser scanning confocal microscope. To quantify the area of protein aggregates and the number of lysotracker or Atg8a-positive dots, grayscale images were converted to binary images (halftone or black & white) with a grayscale cutoff of 20 pixels using ImageJ software [77]. The number/area of positive immunostaining was measured with the “Analyze Particles” function.

### Smox antibody and Western blot

Smox polyclonal antibody was generated against the peptide sequence (DSIVDYPLDNHTHQ) corresponding to amino acids 143–156 (Covance, Dedham, MA, USA) and affinity purified (Thermo/Pierce, Waltham, MA, USA) (specificity documented in Figure S5). Phospho-Smad2 antibody was from Cell Signaling Technology (#3108) (Danvers, MA, USA). Thorax tissue from ten female adults was homogenized in RIPA buffer (Thermo/Pierce, Waltham, MA, USA) with protease inhibitor cocktail (Sigma, St. Louis, MO, USA). Supernatant was incubated with SDS loading buffer (Invitrogen, Grand Island, NY, USA) at 70°C for 10 min. About 30 µg of denatured protein was separated on 10% SDS-polyacrylamide precast gels (Invitrogen Grand Island, NY, USA) and transferred to nitrocellulose membranes. Following incubation with primary and secondary antibodies, the blots were visualized with Pierce ECL Western Blotting Substrate (Thermo Fisher Scientific, Waltham, MA, USA). Band intensity was quantified with Image Lab software (Bio-Rad, Hercules, CA, USA).

### Smox protein production and Electrophoretic Mobility Shift Assays (EMSA)

cDNA for Smox-MH1 DNA binding domain (1–420 nt) was cloned into pFN29K-His6HaloTag protein expression vector (Promega, Madison, WI, USA). After expression in *E. coli*, recombinant proteins were purified using HaloTag purification kit (Promega, Madison, WI, USA). Empty vector was used as a negative control.

Biotin-labeled DNA probes were generated using 3′-end biotin labeling (Fisher/Thermo, Waltham, MA, USA). The binding reactions were carried out in a 10 µl of assay mixture containing 10 mM Tris·HCl (pH 7.5), 150 mM KCl, 5 mM MgCl2, 10 ng/µL poly(dI-dC), ∼50 ng labeled probe and 20 µg purified recombinant protein. After incubation at room temperature for 20 min, the mixtures were electrophoresed on 0.8% agarose gels in 0.5× Tris/borate/EDTA buffer. Biotin-labeled DNAs were transferred to a positive-charged nylon membrane (Invitrogen, Grand Island, NY, USA) and detected using GelShift Chemiluminescent EMSA (Active Motif, Carlsbad, CA, USA).

### Female fecundity

10-day-old mated female flies were maintained on standard food (2% yeast) for five days at three females per vial and 8–10 vials per group. Flies were passed daily to new vials over five days and eggs were counted daily.

### Enzyme Immunoassay (EIA) for hemolymph DILP2

We followed our recently reported EIA assay to measure hemolymph DILP2 [21]. Briefly, about 0.5 µL of hemolymph was collected by decapitation of 15 female flies. Hemolymph was then incubated overnight in a 96-well EIA/RIA plate (Corning Incorporated, Corning, NY, USA) at room temperature. Anti-DILP2 antibody (gift from P. Leopold) was used at 1∶2500 dilution. After the incubation with a HRP-conjugated secondary antibody (1∶2500), hemolymph samples were treated with TMB solution (3,3′,5,5′-teramethylbenzidine; American Qualex antibodies, San Clemente, CA);absorbance was recorded at 450 nm upon a plate reader.

### Statistical analysis

Data are presented as mean ± SEM from three independent biological replicates, unless otherwise noted. Statistical significances were evaluated by t-test and one-way ANOVA analyses using GraphPad Prism Software.

## Supporting Information

Figure S1 Summary and verification of dFOXO ChIP-Seq. (A). The nuclear localization of dFOXO is promoted in *chico ^−/−^* mutants. (B). Binding of dFOXO to *4ebp* promoter is enhanced in *chico* mutants, which is rescued by the removal of dFOXO. Primers for the coding region of Actin gene (*Act5C*) were used as negative control. (C). Pathway analysis of 273 identified dFOXO target genes. (D). Motif analysis on the promoters of dFOXO target genes. (E–F). ChIP-PCR validation of the binding of dFOXO to its target genes in *chico ^−/+^* and IPC ablation mutants. Primers for the coding region of Actin gene (*Act5C*) and *sry* genomic region [70], [78] were used as negative controls. Asterisk indicates significant difference between mutant and wildtype (p<0.05). Three biological replicates were completed for each genotype.
(PDF)

Figure S2 Mortality rate for survival plots of three dFOXO target genes in Figure 1. All UAS-RNAi were driven ubiquitously via GeneSwitch-Gal4 as single genotypes maintained with RU (expressing the RNAi) or without RU (self-control). The natural logarithm of mortality rate is estimated as ln(−ln(1-qx)): qx is age-specific probability of death from census interval x to x+1, calculated as dx/Nx where dx is then observed number of adults dying in the interval x to x+1 and Nx is the number of adults alive at age x. Deaths were recorded across two day census intervals. Mortality rate is not estimated (and thus not plotted) during intervals where no deaths are observed. Aging is slowed by a genotype when it consistently reduces mortality rate across ages where mortality increases as a function of age; this pattern generates divergent survivorship plots with different median lifespans, and produces significance in a log-rank test. We emphasize: significance in a log-rank test alone does not ensure mortality differences are relevant to aging because the test calculates the absolute mortality differential independent of direction, temporal consistency and age-dependence. Likewise, survivorship curves can appear strikingly different among cohorts but not reflect meaningful differences in aging-related mortality. (A) Mortality rate of *daw* RNAi females (RU induced) relative to self-control (no RU) shows strong and consistent reduction. (B) Mortality rate of *Glyp* RNAi females (RU induced) relative to self-control (no RU) shows generally consistent reduction across ages where mortality rate increases with age. (C) Mortality rate of *Tsp42Ef* RNAi females (RU induced) relative to self-control (no RU) shows reduced rate across the final 30 of 40 days where mortality increases with age.
(PDF)

Figure S3 Mortality rate for survival plots of TGF-β pathway in Figure 2. All UAS-RNAi were driven ubiquitously via GeneSwitch-Gal4 as single genotypes maintained with RU (expressing the RNAi) or without RU (self-control). (A) Mortality rate of *dpp* RNAi females (RU induced) relative to self-control (no RU) shows proportionally elevated death across ages where mortality increases with age, suggesting that loss of *dpp* (BMP) in muscle accelerates aging. (B) Muscle specific *daw* RNAi (RU induced) relative to self-control (no RU) shows strong and consistent reduction in mortality, except at oldest ages. (C) Mortality rate of *Mad* RNAi females (RU induced) relative to self-control (no RU) shows elevated death across ages where mortality increases with age, suggesting that loss of *Mad* (BMP) in muscle accelerates aging. (D) Muscle specific *Smox* RNAi (RU induced) relative to self-control (no RU) shows consistent reduction in mortality across intervals where mortality increases with age, although the rate fluctuates at oldest ages.
(PDF)

Figure S4 Mortality rate for survival plots of tissue-specific RNAi of Activin pathway in Figure 3. (A, C, E) Muscle expression via MHC-Gal4; (B, D, F) abdominal fat body expression via S106-GS-Gal4. Reduction of *daw* by RNAi in muscle (A) consistently lowers mortality rate while *daw* RNAi expressed in fat body (B) increases mortality rate. Reduction of *Smox* by RNAi in muscle (C) consistently lowers mortality rate while *Smox* RNAi expressed in fat body (D) increases mortality rate. Reduction of *babo* by RNAi in muscle (E) consistently lowers mortality rate while *babo* RNAi expressed in fat body (B) increases mortality rate at the oldest ages but otherwise does not affect mortality.
(PDF)

Figure S5 Verification of Smox antibody. (A). Smox antibody generated in this study recognizes a 51KDa band that presents in all four tissues tested, which is close to predicted molecular weight for *Drosophila* Smox protein. (B). This Smox antibody can also specifically recognize recombinant Smox proteins expressed in *E. coli*. (C). Fat body-specific knockdown of *Smox* results in reduced protein level visualized with this Smox antibody.
(PDF)

Figure S6 (A–C). Knockdown efficiency of *daw, Smox and babo* RNAi. (D). The comparison of age-dependent climbing activity between control and *daw* RNAi flies. Asterisk indicates significant difference between treatment and control (*p*<0.05). (E). dFOXO shows less binding to the promoter of *Atg8a*. (F). Fat body-specific *daw* RNAi increased the level of circulating DILP2.
(PDF)

Figure S7 Mortality rate for survival plots of the impact of Atg8a upon aging, text Figure 7. (A) Over expression of *Atg8a* in muscle by the constitutive driver MHC-gal4 consistently reduces mortality rate across intervals where the death rate increases with age. There is high variability in mortality rate after age 70 days including an interval where no deaths were observed in the MHC; UAS-*Atg8a* genotype. (B) Mortality rate for survival plots of the genetic epistasis between *daw* and *Atg8a*. Reducing *daw* by RNAi lowers mortality rate relative to wildtype control after age 40 days. The net survival benefit of *daw* RNAi in this trial is muted because this cohort shows somewhat high mortality rate across the early intervals when death rate does not yet increase with age in the control cohort. *Atg8a* RNAi alone shows nearly identical mortality rate as control. Mortality rate of the *daw* and *Atg8a* RNAi double is similar to or slightly greater than that of control, indicating that *Atg8a* RNAi rescues the mortality benefit conferred by *daw* RNAi. Statistics of Cox proportional hazard for this epistasis are presented in Table S6.
(PDF)

Figure S8 Proposed model for the autonomous and non-autonomous roles of Activin signaling combine to control aging. Activin signaling targeted by insulin/dFOXO negatively regulates muscle autophagy, protein homeostasis and muscle functions in a cell autonomous manner, while it could also nonautonomously modulate longevity, the secretion of insulin peptides from the brain and peripheral insulin signaling. InR: insulin receptor; IPC: insulin producing cells. Myokine refers to unknown muscle-derived hormonal factor.
(PDF)

Table S1 Summary of lifespan analyses for 23 dFOXO-target genes reduced by RNAi.
(DOCX)

Table S2 Summary of lifespan analyses for seven dFOXO-target genes increased by over-expression.
(DOCX)

Table S3 Summary of lifespan analyses for Drosophila TGF-β pathway.
(DOCX)

Table S4 Summary of lifespan analyses for tissue-specific knockdown of Activin signaling and of *Atg8a* over-expression.
(DOCX)

Table S5 Mean lifespan and sample size of muscle-specific knockdown of *dawdle* and *Atg8a*.
(PDF)

Table S6 Cox proportional hazard survival analysis for the effects of muscle-specific knockdown of *dawdle* and *Atg8a*.
(PDF)

Table S7 dFOXO direct target genes in *chico* and IPC ablation mutants.
(XLSX)

Table S8 Primers for mRNA analyses.
(XLSX)
