# Supplementary figures and images for: Activin Signaling Targeted by Insulin/dFOXO Regulates Aging and Muscle Proteostasis in Drosophila
Source: PLoS Genet. 2013 Nov 7;9(11):e1003941. doi: 10.1371/journal.pgen.1003941 (PMC3820802; doi:10.1371/journal.pgen.1003941)

Figure S1

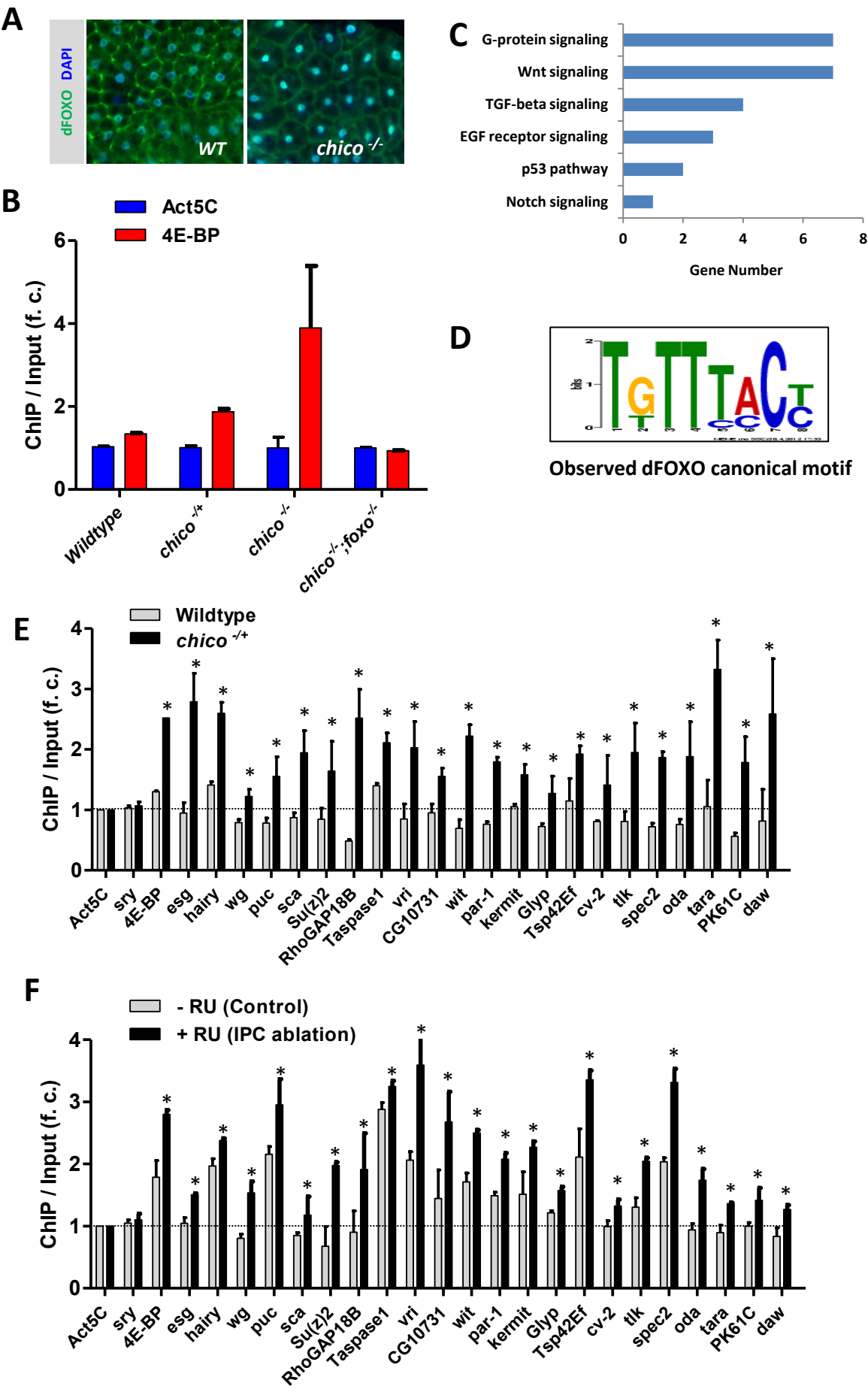

Supplement: Figure S1 — Summary and verification of dFOXO ChIP-Seq. (A). The nuclear localization of dFOXO is promoted in chico −/− mutants. (B). Binding of dFOXO to 4ebp promoter is enhanced in chico mutants, which is rescued by the removal of dFOXO. Primers for the coding region of Actin gene (Act5C) were used as negative control. (C). Pathway analysis of 273 identified dFOXO target genes. (D). Motif analysis on the promoters of dFOXO target genes. (E–F). ChIP-PCR validation of the binding of dFOXO to its target genes in chico −/+ and IPC ablation mutants. Primers for the coding region of Actin gene (Act5C) and sry genomic region [70], [78] were used as negative controls. Asterisk indicates significant difference between mutant and wildtype (p<0.05). Three biological replicates were completed for each genotype. (PDF) [file pgen.1003941.s001.pdf]

Figure S2

Screen of dFOXO target genes

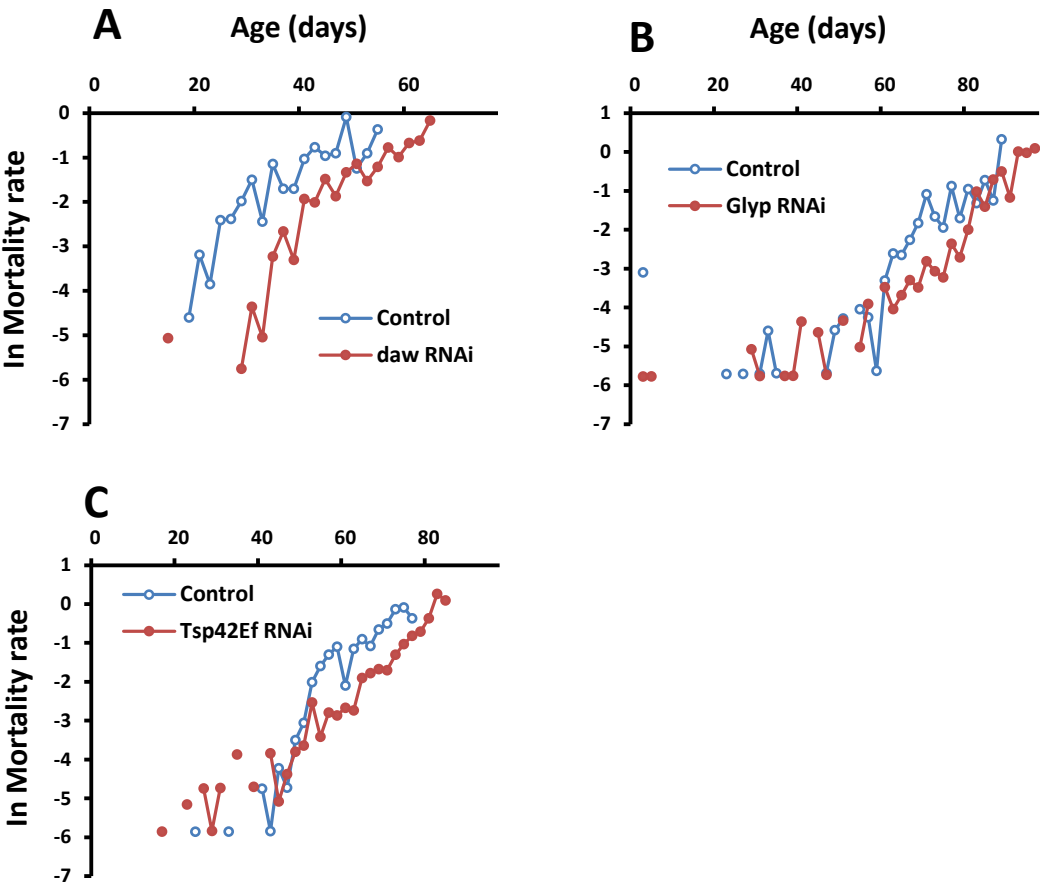

Supplement: Figure S2 — Mortality rate for survival plots of three dFOXO target genes in Figure 1. All UAS-RNAi were driven ubiquitously via GeneSwitch-Gal4 as single genotypes maintained with RU (expressing the RNAi) or without RU (self-control). The natural logarithm of mortality rate is estimated as ln(−ln(1-qx)): qx is age-specific probability of death from census interval x to x+1, calculated as dx/Nx where dx is then observed number of adults dying in the interval x to x+1 and Nx is the number of adults alive at age x. Deaths were recorded across two day census intervals. Mortality rate is not estimated (and thus not plotted) during intervals where no deaths are observed. Aging is slowed by a genotype when it consistently reduces mortality rate across ages where mortality increases as a function of age; this pattern generates divergent survivorship plots with different median lifespans, and produces significance in a log-rank test. We emphasize: significance in a log-rank test alone does not ensure mortality differences are relevant to aging because the test calculates the absolute mortality differential independent of direction, temporal consistency and age-dependence. Likewise, survivorship curves can appear strikingly different among cohorts but not reflect meaningful differences in aging-related mortality. (A) Mortality rate of daw RNAi females (RU induced) relative to self-control (no RU) shows strong and consistent reduction. (B) Mortality rate of Glyp RNAi females (RU induced) relative to self-control (no RU) shows generally consistent reduction across ages where mortality rate increases with age. (C) Mortality rate of Tsp42Ef RNAi females (RU induced) relative to self-control (no RU) shows reduced rate across the final 30 of 40 days where mortality increases with age. (PDF) [file pgen.1003941.s002.pdf]

Figure S3

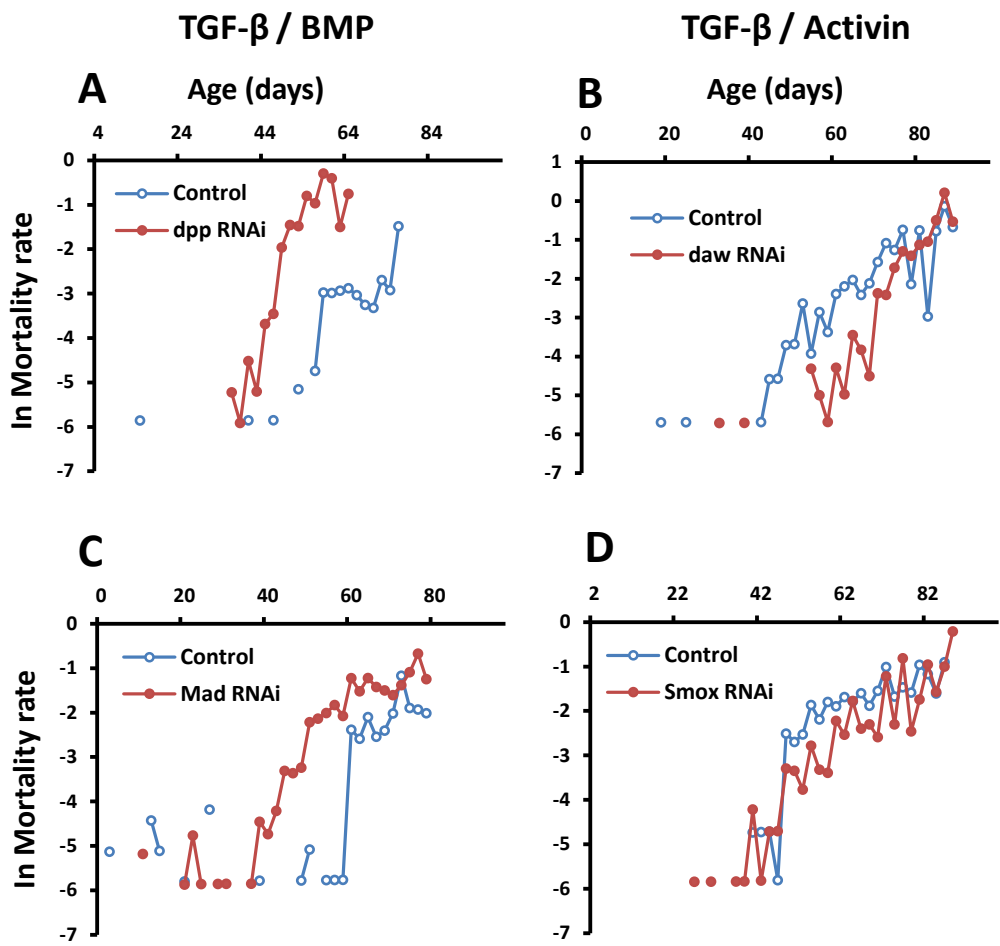

Supplement: Figure S3 — Mortality rate for survival plots of TGF-β pathway in Figure 2. All UAS-RNAi were driven ubiquitously via GeneSwitch-Gal4 as single genotypes maintained with RU (expressing the RNAi) or without RU (self-control). (A) Mortality rate of dpp RNAi females (RU induced) relative to self-control (no RU) shows proportionally elevated death across ages where mortality increases with age, suggesting that loss of dpp (BMP) in muscle accelerates aging. (B) Muscle specific daw RNAi (RU induced) relative to self-control (no RU) shows strong and consistent reduction in mortality, except at oldest ages. (C) Mortality rate of Mad RNAi females (RU induced) relative to self-control (no RU) shows elevated death across ages where mortality increases with age, suggesting that loss of Mad (BMP) in muscle accelerates aging. (D) Muscle specific Smox RNAi (RU induced) relative to self-control (no RU) shows consistent reduction in mortality across intervals where mortality increases with age, although the rate fluctuates at oldest ages. (PDF) [file pgen.1003941.s003.pdf]

Figure S4

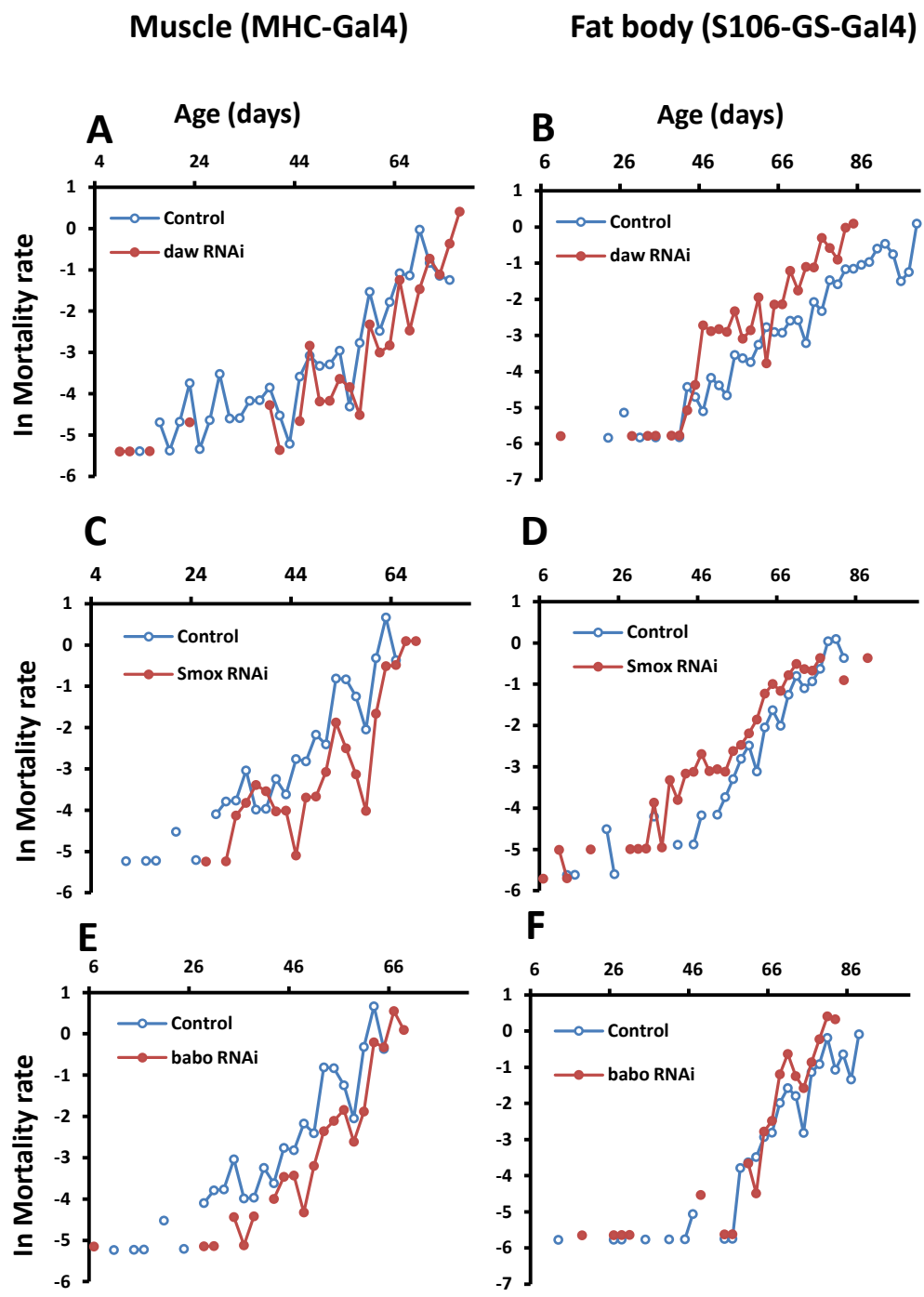

Supplement: Figure S4 — Mortality rate for survival plots of tissue-specific RNAi of Activin pathway in Figure 3. (A, C, E) Muscle expression via MHC-Gal4; (B, D, F) abdominal fat body expression via S106-GS-Gal4. Reduction of daw by RNAi in muscle (A) consistently lowers mortality rate while daw RNAi expressed in fat body (B) increases mortality rate. Reduction of Smox by RNAi in muscle (C) consistently lowers mortality rate while Smox RNAi expressed in fat body (D) increases mortality rate. Reduction of babo by RNAi in muscle (E) consistently lowers mortality rate while babo RNAi expressed in fat body (B) increases mortality rate at the oldest ages but otherwise does not affect mortality. (PDF) [file pgen.1003941.s004.pdf]

Figure S5

A

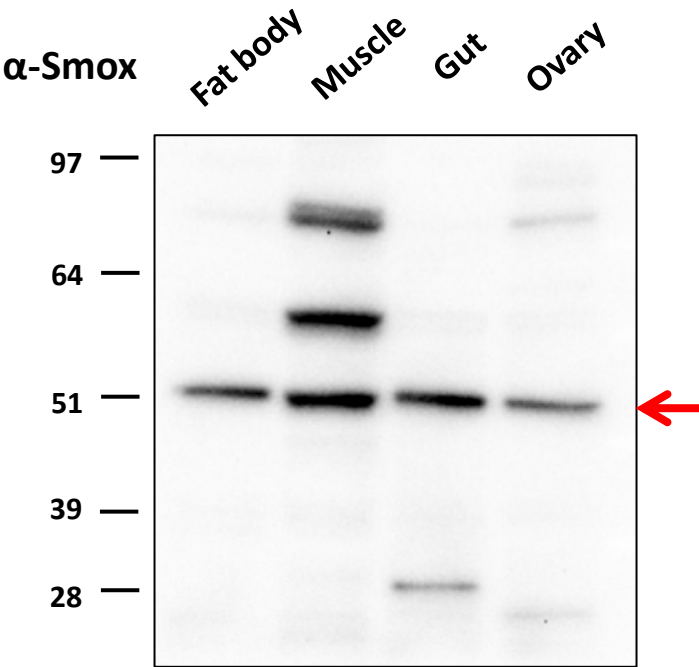

B

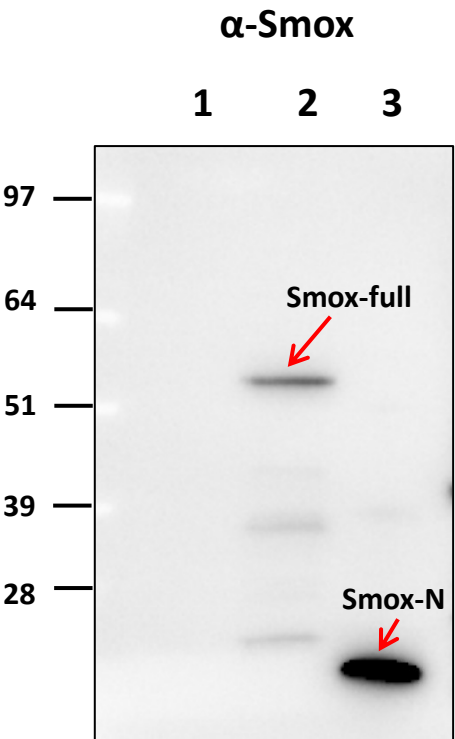

1: Vector  
2: Smox-Full  
3: Smox-N-term

C

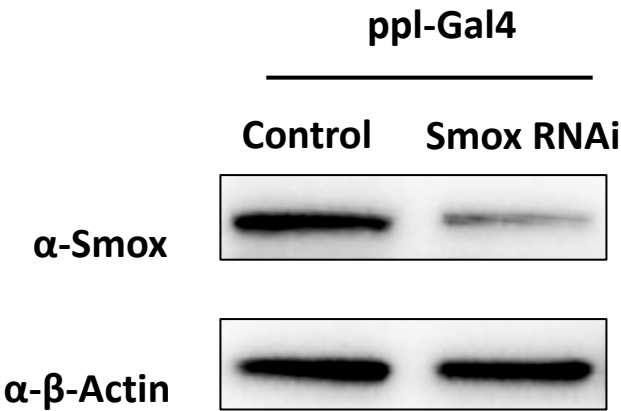

Supplement: Figure S5 — Verification of Smox antibody. (A). Smox antibody generated in this study recognizes a 51KDa band that presents in all four tissues tested, which is close to predicted molecular weight for Drosophila Smox protein. (B). This Smox antibody can also specifically recognize recombinant Smox proteins expressed in E. coli. (C). Fat body-specific knockdown of Smox results in reduced protein level visualized with this Smox antibody. (PDF) [file pgen.1003941.s005.pdf]

Figure S6

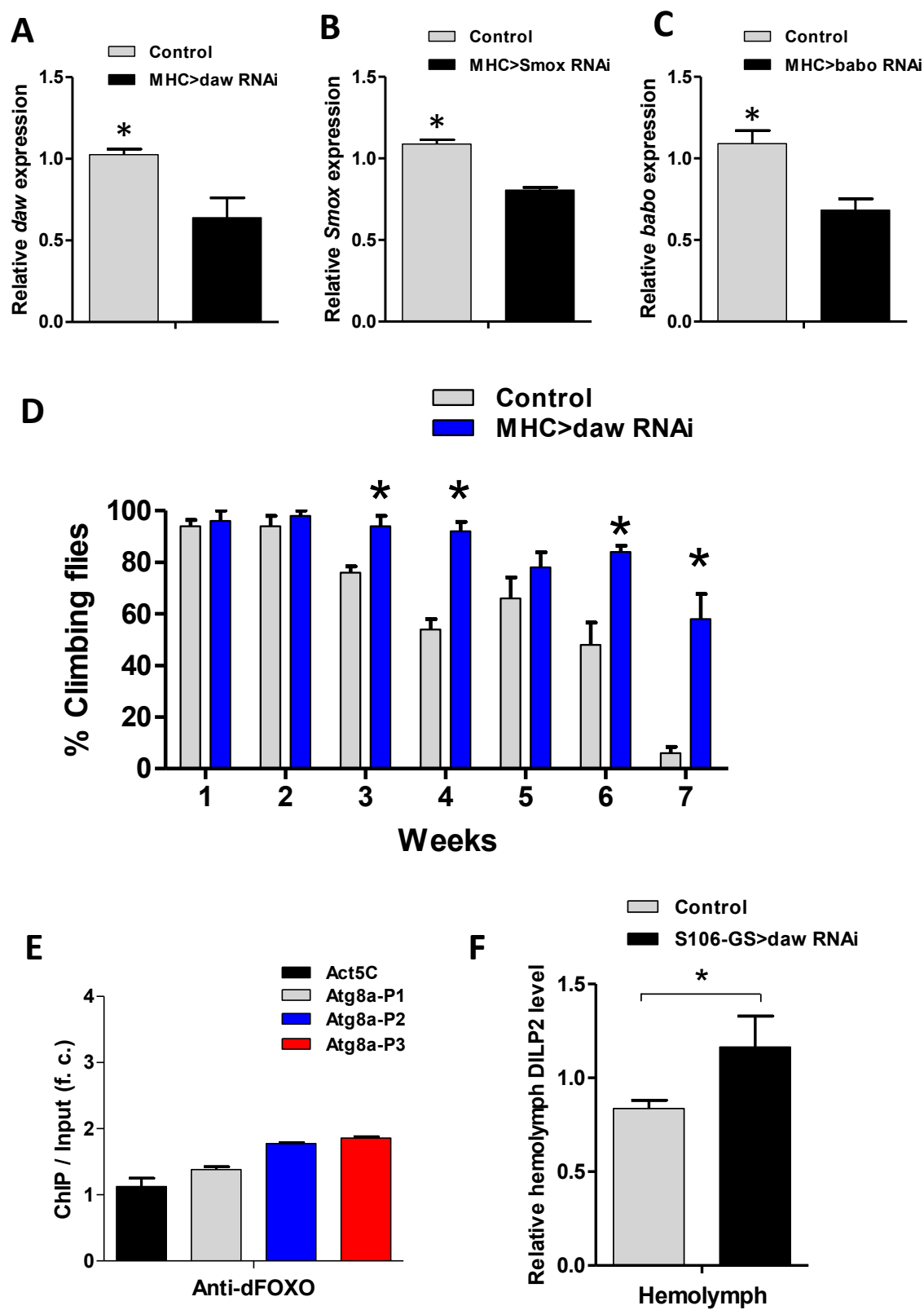

Supplement: Figure S6 — (A–C). Knockdown efficiency of daw, Smox and babo RNAi. (D). The comparison of age-dependent climbing activity between control and daw RNAi flies. Asterisk indicates significant difference between treatment and control (p<0.05). (E). dFOXO shows less binding to the promoter of Atg8a. (F). Fat body-specific daw RNAi increased the level of circulating DILP2. (PDF) [file pgen.1003941.s006.pdf]

Figure S7

A

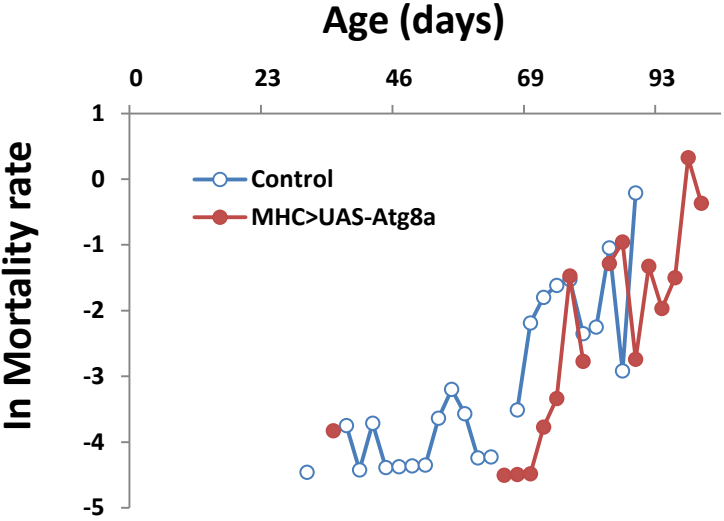

B

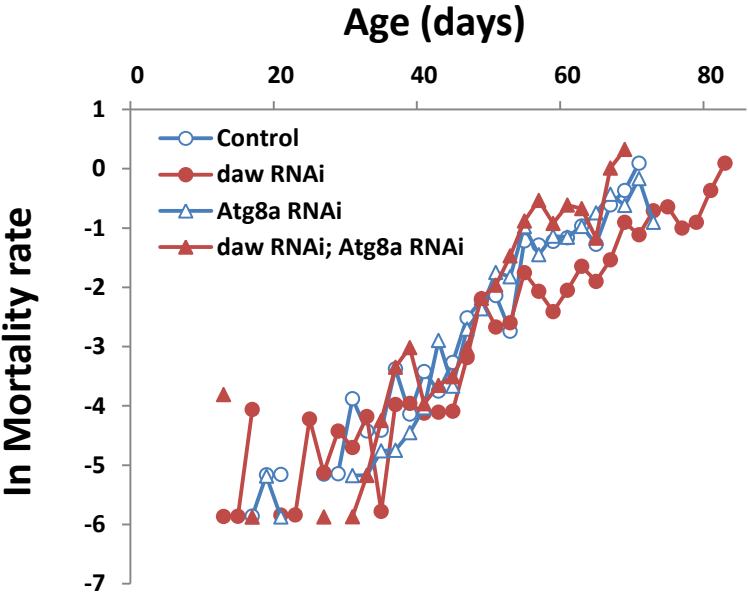

Supplement: Figure S7 — Mortality rate for survival plots of the impact of Atg8a upon aging, text Figure 7. (A) Over expression of Atg8a in muscle by the constitutive driver MHC-gal4 consistently reduces mortality rate across intervals where the death rate increases with age. There is high variability in mortality rate after age 70 days including an interval where no deaths were observed in the MHC; UAS-Atg8a genotype. (B) Mortality rate for survival plots of the genetic epistasis between daw and Atg8a. Reducing daw by RNAi lowers mortality rate relative to wildtype control after age 40 days. The net survival benefit of daw RNAi in this trial is muted because this cohort shows somewhat high mortality rate across the early intervals when death rate does not yet increase with age in the control cohort. Atg8a RNAi alone shows nearly identical mortality rate as control. Mortality rate of the daw and Atg8a RNAi double is similar to or slightly greater than that of control, indicating that Atg8a RNAi rescues the mortality benefit conferred by daw RNAi. Statistics of Cox proportional hazard for this epistasis are presented in Table S6. (PDF) [file pgen.1003941.s007.pdf]

Figure S8

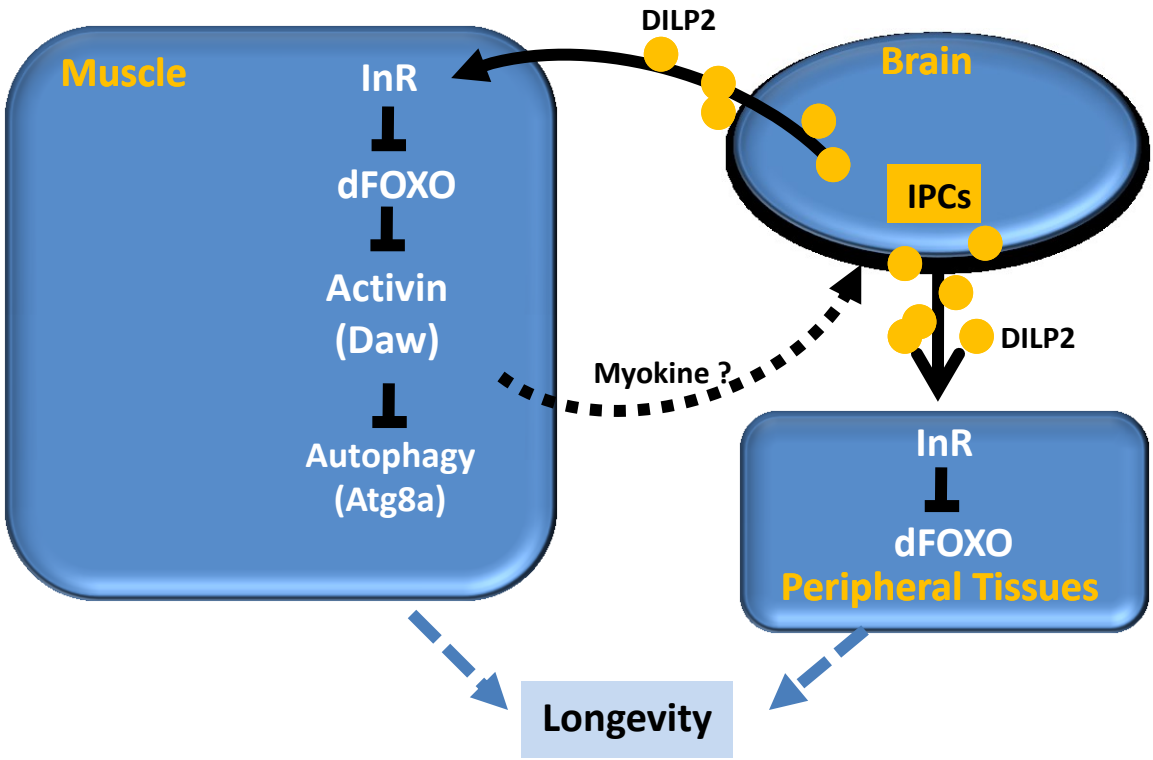

Supplement: Figure S8 — Proposed model for the autonomous and non-autonomous roles of Activin signaling combine to control aging. Activin signaling targeted by insulin/dFOXO negatively regulates muscle autophagy, protein homeostasis and muscle functions in a cell autonomous manner, while it could also nonautonomously modulate longevity, the secretion of insulin peptides from the brain and peripheral insulin signaling. InR: insulin receptor; IPC: insulin producing cells. Myokine refers to unknown muscle-derived hormonal factor. (PDF) [file pgen.1003941.s008.pdf]
