# Supplementary material for: Activin Signaling Targeted by Insulin/dFOXO Regulates Aging and Muscle Proteostasis in Drosophila
Source: PLoS Genet. 2013 Nov 7;9(11):e1003941. doi: 10.1371/journal.pgen.1003941 (PMC3820802; doi:10.1371/journal.pgen.1003941)
Supplement: Table S1 — Summary of lifespan analyses for 23 dFOXO-target genes reduced by RNAi. (DOCX) [file pgen.1003941.s009.docx]

**Table S1. Summary of lifespan analysis on 23 dFOXO target genes via RNAi**

|  | **Gene name** | **Driver** | **RNAi line** | **Mean lifespan (E0, d)** | | | **E0  dif.** | **Prob.**  **(Log-rank)** | **Sample size** |
| --- | --- | --- | --- | --- | --- | --- | --- | --- | --- |
|  |  |  |  | **Control** | **RNAi** | | **(%)** |  | **(No. flies)** |
| Activator | GlyP | ^3^Tub-GS-dicer2 | ^1^BL33634 | 70 | 82 | | 17.1 | <.0001 | 640 |
|  | cv-2 | Tub-GS-dicer2 | ^2^VDRC109915 | 59 | 67 | | 13.6 | <.0001 | 602 |
|  | puc | Tub-GS | BL31557 | 65.6 | 65.4 | | -0.3 | 0.0255 | 675 |
|  | sca | Tub-GS-dicer2 | BL28675 | 69 | 67 | | -2.9 | 0.0006 | 663 |
|  | RhoGAP18B | Tub-GS | BL31165 | 62 | 69 | | 11.3 | 0.0017 | 173 |
|  | Su(z)2 | Tub-GS-dicer2 | BL33403 | 66.4 | 65.9 | | -0.75 | 0.0843 | 265 |
|  | vri | Tub-GS | BL25989 | 84 | 82 | | -2.4 | 0.8626 | 735 |
|  | wg | Tub-GS | BL32994 | 74 | 70 | | -5.4 | <.0001 | 665 |
|  | wit | Tub-GS | BL25949 | 64 | 60 | | -6.3 | 0.0017 | 636 |
|  | h | Tub-GS | BL27738 | 76 | 64 | | -15.8 | <.0001 | 715 |
|  | tara | Tub-GS | BL31634 | 66 | 54 | | -18.2 | <.0001 | 650 |
|  | 4ebp | Not tested | | | | | | | |
| Repressor | daw | Tub-GS | BL34974 | 34 | 46 | 35.3 | | <.0001 | 524 |
|  | tlk | Tub-GS | BL33983 | 73 | 75 | 2.7 | | 0.9239 | 692 |
|  | Spec2 | Tub-GS-dicer2 | VDRC101359 | 72 | 70 | -2.8 | | <.0001 | 656 |
|  | esg | Tub-GS | BL28514 | 76 | 72 | -5.3 | | <.0001 | 711 |
|  | CG10731 | Tub-GS-dicer2 | VDRC109949 | 74 | 68 | -8.1 | | <.0001 | 680 |
|  | Taspase1 | Tub-GS-dicer2 | VDRC110147 | 66 | 46 | -30.3 | | <.0001 | 720 |
|  | par-1 | Tub-GS | BL32410 | 36 | 14 | -61.1 | | <.0001 | 681 |
| Others | Tsp42Ef | Tub-GS-dicer2 | VDRC8712 | 55 | 65 | 18.2 | | <.0001 | 699 |
|  | kermit | Tub-GS | VDRC109297 | 63 | 61 | -3.2 | | <.0001 | 680 |
|  | Oda | Tub-GS | BL35436 | 70 | 66 | -5.7 | | <.0001 | 161 |
|  | PK61C | Tub-GS-dicer2 | BL27725 | 60 | 44 | -26.7 | | <.0001 | 670 |

1. BL lines are from Bloomington Drosophila Stock Center.

2. VDRC lines are from Vienna Drosophila RNAi Center.

3. Tub-GS-dicer2: Ubiquitous tubulin GeneSwitch (GS)-Gal4 driver contains UAS-dicer2 to enhance the knockdown.

4. Probability is based on the log-rank test for net differences in mortality rate. Note that when survivorship curves ‘cross-over’ it is possible to have find cohorts with similar median life expectancy but significant differences in mortality because the relative mortality benefit at ages before the median are balanced by a mortality deficit at later ages.
