# Supplementary material for: Activin Signaling Targeted by Insulin/dFOXO Regulates Aging and Muscle Proteostasis in Drosophila
Source: PLoS Genet. 2013 Nov 7;9(11):e1003941. doi: 10.1371/journal.pgen.1003941 (PMC3820802; doi:10.1371/journal.pgen.1003941)
Supplement: Table S2 — Summary of lifespan analyses for seven dFOXO-target genes increased by over-expression. (DOCX) [file pgen.1003941.s010.docx]

**Table S2. Summary of lifespan analysis on seven dFOXO target genes via over-expression**

|  | **Gene name** | **Driver** | **Overexpression line** | **Mean lifespan (E0, d)** | | **E0  dif.** | **Prob.**  **(Log-rank)** | **Sample size** |
| --- | --- | --- | --- | --- | --- | --- | --- | --- |
|  |  |  |  | **Control** | **Overexpression** | **(%)** |  | **(No. flies)** |
| Activator | h | Tub-GS | UAS-hairy | 74 | 58 | -21.6 | <.0001 | 703 |
|  | wg | da-GS | ^1^BL5919 | 68 | 54 | -20.6 | <.0001 | 716 |
|  | puc | da-GS | UAS-puc | 54 | 30 | -44.44 | <.0001 | 705 |
|  | RhoGAP18B | Tub-GS | UAS-RhoGAP18B | 70 | 68 | -2.9 | 0.1072 | 596 |
|  | vri | Tub-GS | UAS-vri | 70 | 72 | 2.9 | 0.0005 | 589 |
|  | wit | Tub-GS | UAS-wit | 70 | 42 | -40 | <.0001 | 671 |
|  | cv-2 | da-GS | UAS-CV-2 | 64 | 62 | -3.1 | <.0001 | 670 |

1. BL5919 line is from Bloomington Drosophila Stock Center. Other lines are lab collections (see Experimental Procedures).
2. Probability is based on the log-rank test for net differences in mortality rate. Note that when survivorship curves ‘cross-over’ it is possible to have find cohorts with similar median life expectancy but significant differences in mortality because the relative mortality benefit at ages before the median are balanced by a mortality deficit at later ages
