# Supplementary material for: Activin Signaling Targeted by Insulin/dFOXO Regulates Aging and Muscle Proteostasis in Drosophila
Source: PLoS Genet. 2013 Nov 7;9(11):e1003941. doi: 10.1371/journal.pgen.1003941 (PMC3820802; doi:10.1371/journal.pgen.1003941)
Supplement: Table S3 — Summary of lifespan analyses for Drosophila TGF-β pathway. (DOCX) [file pgen.1003941.s011.docx]

**Table S3. Summary of lifespan analysis on *Drosophila* TGF-β pathway**

|  | | **Gene name** | **Driver** | **RNAi line** | **Mean lifespan (E0, d)** | | **E0  dif.** | **Prob.**  **(Log-rank )** | **Sample size** |
| --- | --- | --- | --- | --- | --- | --- | --- | --- | --- |
|  |  |  |  |  | **Control** | **RNAi** | **(%)** |  | **(No. flies)** |
| Activin subfamily | Ligand | Daw | da-GS | ^1^BL34974 | 68 | 76 | 11.8 | <.0001 | 601 |
|  |  |  | Tub-GS | BL34974 | 34 | 46 | 35.3 | <.0001 | 524 |
|  | Ligand | Act-β | da-GS | BL29597 | 70 | 68 | -2.9 | <.0001 | 705 |
|  | Type I Receptor | Babo | da-GS | BL25933 | 75.2 | 76.9 | 2.3 | <.0001 | 713 |
|  | R-Smad | Smox | Tub-GS | BL26756 | 60 | 66 | 10.0 | <.0001 | 689 |
| BMP subfamily | Ligand | Dpp | da-GS | BL33618 | 76 | 54 | -28.9 | <.0001 | 722 |
|  | Ligand | Gbb | da-GS | BL34898 | 76 | 62 | -18.4 | <.0001 | 719 |
|  | Ligand | Scw | Not tested | | | | | | |
|  | Type I Receptor | Tkv | Tub-GS | BL31041 | 68 | 64 | -5.9 | 0.6929 | 678 |
|  | Type I Receptor | Sax | da-GS | BL36131 | 54 | 56 | 3.7 | 0.1267 | 581 |
|  | R-Smad | Mad | Tub-GS | BL31315 | 72 | 56 | -22.2 | <.0001 | 696 |
| Shared | Co-Smad | Med | Tub-GS | BL31928 | 68 | 48 | -29.4 | <.0001 | 594 |
|  | Type II Receptor | Punt | da-GS | BL27514 | 74 | 66 | -10.8 | <.0001 | 707 |
|  | Type II Receptor | Wit | da-GS | BL25949 | 64 | 60 | -6.3 | 0.0017 | 636 |
| Other TGF- β ligands | Ligand | Myo | Tub-GS | BL31200 | 76 | 70 | -7.9 | <.0001 | 583 |
|  | Ligand | Mav | da-GS | BL34650 | 72 | 60 | -16.7 | <.0001 | 689 |

1. BL lines are from Bloomington Drosophila Stock Center.
2. Probability is based on the log-rank test for net differences in mortality rate. Note that when survivorship curves ‘cross-over’ it is possible to have find cohorts with similar median life expectancy but significant differences in mortality because the relative mortality benefit at ages before the median are balanced by a mortality deficit at later ages.
