# Supplementary material for: Activin Signaling Targeted by Insulin/dFOXO Regulates Aging and Muscle Proteostasis in Drosophila
Source: PLoS Genet. 2013 Nov 7;9(11):e1003941. doi: 10.1371/journal.pgen.1003941 (PMC3820802; doi:10.1371/journal.pgen.1003941)
Supplement: Table S4 — Summary of lifespan analyses for tissue-specific knockdown of Activin signaling and of Atg8a over-expression. (DOCX) [file pgen.1003941.s012.docx]

**Table S4. Summary of lifespan analysis on tissue-specific knockdown of Activin signaling and over-expressing autophagy gene, Atg8a**

| **Tissue** | **Gene name** | **Driver** | **RNAi or overexpression**  **line** | **Mean lifespan**  **(E0, d)** | | **E0  dif.** | **Prob.**  **(Log-Rank)** | **Sample size** |
| --- | --- | --- | --- | --- | --- | --- | --- | --- |
|  |  |  |  | **Control** | **RNAi** | **(%)** |  | **(No. flies)** |
| Muscle | Daw | MHC-Gal4 | ^1^VDRC105309-First trial | 74 | 82 | 10.81 | <.0001 | 422 |
|  |  |  | ^1^VDRC105309-Seconde trial | 69.9 | 77.6 | 11.02 | <.0001 | 243 |
|  |  |  | ^2^BL34974 | 62 | 69 | 11.3 | <.0001 | 360 |
|  | Babo | MHC-Gal4 | BL25933 | 61 | 71 | 16.39 | <.0001 | 361 |
|  | Smox | MHC-Gal4 | BL26756 | 61 | 71 | 16.39 | <.0001 | 375 |
|  | Atg8a | MHC-Gal4 | BL37750 | 73 | 83 | 13.7 | <.0001 | 274 |
| Fat body | Daw | S106-GS | BL34974 | 78 | 66 | -15.38 | <.0001 | 668 |
|  | Babo | S106-GS | BL25933 | 74 | 72 | -2.70 | <.0001 | 608 |
|  | Smox | S106-GS | BL26756 | 66 | 60 | -9.09 | <.0001 | 579 |

1. VDRC lines are from Vienna Drosophila RNAi Center.

2. BL lines are from Bloomington Drosophila Stock Center.

3. Probability is based on the log-rank test for net differences in mortality rate. Note that when survivorship curves ‘cross-over’ it is possible to have find cohorts with similar median life expectancy but significant differences in mortality because the relative mortality benefit at ages before the median are balanced by a mortality deficit at later ages.
