# Supplementary material for: Activin Signaling Targeted by Insulin/dFOXO Regulates Aging and Muscle Proteostasis in Drosophila
Source: PLoS Genet. 2013 Nov 7;9(11):e1003941. doi: 10.1371/journal.pgen.1003941 (PMC3820802; doi:10.1371/journal.pgen.1003941)
Supplement: Table S5 — Mean lifespan and sample size of muscle-specific knockdown of dawdle and Atg8a. (PDF) [file pgen.1003941.s013.pdf]

Table S5. Mean lifespan and sample size of muscle-specific knockdown of dawdle and Atg8a.

Genotypes	RNAi line	Mean lifespan (days)	Sample size (No. flies)
MHC-Gal4/+	/	52	351
MHC-Gal4/Daw RNAi	VDRC105309	55.2	353
MHC-Gal4/Atg8a RNAi	BL34340	52.8	358
MHC-Gal4/Daw RNAi; Atg8a RNAi	VDRC105309, BL34340	50.5	365
