# Supplementary material for: Activin Signaling Targeted by Insulin/dFOXO Regulates Aging and Muscle Proteostasis in Drosophila
Source: PLoS Genet. 2013 Nov 7;9(11):e1003941. doi: 10.1371/journal.pgen.1003941 (PMC3820802; doi:10.1371/journal.pgen.1003941)
Supplement: Table S6 — Cox proportional hazard survival analysis for the effects of muscle-specific knockdown of dawdle and Atg8a. (PDF) [file pgen.1003941.s014.pdf]

Table S6. Cox proportional hazard survival analysis for the effects of muscle-specific knockdown of *dawdle* and *Atg8a* on lifespan. β_i is the estimate of model parameter and CL is confidence limit (significant when the interval does not bound zero). The hazard ratio is exp(β_i) and indicates similar mortality to wildtype when 1.0, lower mortality when less then 1 and higher mortality when greater than 1. *Daw* RNAi reduces mortality about 35%, *Atg8a* RNAi has no significant effect on mortality, the double RNAi genotype has 34% elevated mortality and thereby reverses the benefit of *Daw* RNAi.

Model effect	β_i	Std Error	Lower CL	Upper CL	Hazard ratio
Daw RNAi	-0.438	0.0494	-0.5363	-0.3426	0.645
Atg8a RNAi	0.089	0.0461	-0.0022	0.1784	1.093
Daw RNAi; Atg8a RNAi	0.294	0.0467	0.2014	0.3846	1.342
